# Proteomic profile of hippocampal growth cones through early postnatal development

**DOI:** 10.1242/dev.205342

**Published:** 2026-06-22

**Authors:** Maike Krause, Kamilla Aase Kronberg, Paulo J. B. Girão, Lara Sophie Strohmeier, Giulia Quattrocolo

**Affiliations:** ^1^ Kavli Institute for Systems Neuroscience and Centre for Algorithms of the Cortex, Norwegian University of Science and Technology (NTNU), 7491 Trondheim, Norway; ^2^ Mohn Research Center for the Brain, Norwegian University of Science and Technology (NTNU), 7491 Trondheim, Norway

**Keywords:** Early postnatal development, Mouse, Growth cones, Proteomics, Local translation, Hippocampus

## Abstract

Axonal growth cones rely on local proteomic changes to interpret guidance cues while navigating towards their synaptic targets. Yet the developmental dynamics of their proteome remain poorly understood. By isolating growth cones from the whole mouse hippocampus and dentate gyrus at early postnatal days, we performed a systematic characterization of their proteome. We show that growth cones from both subregions share a proteomic signature at postnatal day (P) 1, dominated by energy metabolism and protein synthesis, before taking distinct developmental trajectories. In fact, hippocampal growth cones undergo a rapid transition, from RNA-related processes to synapse formation. Dentate gyrus growth cones, however, maintain a similar proteomic profile at P3 and P5, suggesting a prolonged exploratory behaviour, consistent with later ingrowth of entorhinal cortex fibres. At these early timepoints, hippocampal and dentate gyrus growth cones are characterized by several temporally regulated proteins, suggesting dynamic and different developmental trajectories. Our dataset provides a comprehensive proteomic resource for understanding growth cone function during hippocampal circuit formation and insights into the molecular mechanisms underlying region-specific developmental timelines.

## Teaser

**Summary:** Systematic proteomic analysis of early postnatal hippocampal growth cones highlights subregion-specific developmental trajectories. Hippocampal growth cones transition faster to more mature stages compared to growth cones from the dentate gyrus.

## INTRODUCTION

The formation of precise neural connections during development depends on the ability of growing axons to navigate complex environments and establish synaptic contacts with their target cells. This process is mediated by growth cones (GCs), highly dynamic structures located at the tips of extending axons (Goodman and Shatz, 1993; Ramón y Cajal, 1890). GCs integrate multiple guidance cues, including attractive and repulsive signals, to direct axon pathfinding toward specific targets while avoiding unspecific connections. The molecular machinery involved in the GC movement are complex signalling cascades that link extracellular guidance cues to cytoskeletal rearrangements (Dickson, 2002; Govek et al., 2005; Luo, 2000; Pérez-Ferrer and Herrera, 2025; Soriano et al., 2021; Tessier-Lavigne and Goodman, 1996). GC responses are highly context-dependent and can be modulated by the developmental stage of the neuron, the local molecular environment and the activity state of the GC itself (Koppers and Holt, 2022). These complex and dynamic processes that the GCs undergo are reflected in the locally expressed proteins. *In vitro* and *in vivo* studies have highlighted the ability of GCs to quickly respond to changes in the encountered cues, regulating both transcriptomic and proteomic content (Cagnetta et al., 2018; Koppers et al., 2019; Pérez-Ferrer and Herrera, 2025; Shigeoka et al., 2016). However, thorough developmental analyses of the GC proteome *in vivo* are limited (Cagnetta et al., 2018; Chauhan et al., 2020; Dumrongprechachan et al., 2022; Estrada-Bernal et al., 2012; Nozumi et al., 2009; Poulopoulos et al., 2019). Here, we focus on hippocampal GCs (HP-GCs), analysing proteomic changes within a short but critical developmental window.

The hippocampal circuit is one of the most extensively studied neural networks in the mammalian brain (Moser et al., 2008). This circuit comprises a complex network of interconnected structures, including the entorhinal cortex (EC) and hippocampus (HP) with hippocampal subfields cornu ammonis (CA) 1 to CA3 and the dentate gyrus (DG) (Amaral and Witter, 1989). Entorhinal-hippocampal connectivity follows a precise temporal sequence, with major projection pathways established during embryonic and early postnatal periods in rodents (Amaral and Dent, 1981; Del Río et al., 1997; Fricke and Cowan, 1977; Supèr and Soriano, 1994; Witter et al., 2017). Reelin-positive Layer 2 neurons, the major input to the HP, project primarily to the outer part of the molecular layer (ML) of the DG and to the stratum lacunosum-moleculare (SLM) of CA3, while Layer 3 neurons predominantly target CA1 and the subicular complex (Steward and Scoville, 1976; Witter et al., 1988). In parallel, intrahippocampal connectivity develops. DG cells extend mossy fibres toward CA3 soon after birth (Amaral and Dent, 1981; Blackstad et al., 1970) and, between postnatal day (P) 2 and P5, CA3 pyramidal cells form Schaffer collaterals connecting to CA1. This laminar specificity is achieved through precise axon guidance mechanisms and activity-dependent refinement processes (Canto et al., 2008; Förster et al., 2006; Mata et al., 2018).

Hippocampal invasion by its major input cells requires precise coordination of attractive and repulsive guidance signals, with molecules such as reelin, slit proteins and semaphorins playing crucial roles in directing axon pathfinding (Del Río et al., 1997; Förster et al., 2002; Mata et al., 2018). How these changes are dynamically represented in HP-GCs is unknown. To address this question, we performed systematic proteomic profiling of GCs from the whole HP and the DG across multiple timepoints. Such an approach has not yet been achieved, with earlier studies mainly providing proteomic insights at single developmental timepoints of subcellular fractions from whole brain or forebrain tissue, or examining subcellular-specific GC proteomes (Chauhan et al., 2020; Estrada-Bernal et al., 2012; Nozumi et al., 2009; Poulopoulos et al., 2019). Moreover, recent advances in understanding post-transcriptional and translational regulation during brain development have highlighted how local protein synthesis shapes neuronal identity and connectivity (Borisova et al., 2024; Harnett et al., 2022; Veeraraghavan et al., 2026). Studying the local GC proteome can provide further insights into how circuit development is locally regulated, serving as a functional readout for mRNA localization and local translation.

Importantly, many of the molecular pathways active during the development of circuits have been linked to neurological disorders and neurodegenerative diseases. Disruptions in GC function, axon guidance and local protein syntheses have been implicated in conditions ranging from autism spectrum disorders to amyotrophic lateral sclerosis and epilepsy (Parenti et al., 2020; Weerasinghe-Mudiyanselage et al., 2022). A better understanding of GC proteomic composition during critical developmental windows may therefore provide insights into the developmental origin of such pathologies.

Layer 2 EC fibres are the major input to the DG and CA3 regions. Thus, we first characterized the ingrowth timeline of those fibres by using a recently developed cell-type-specific mouse line (Blankvoort et al., 2018). This analysis helped us to narrow down three crucial timepoints (P1, P3 and P5) for isolating GCs from the HP (HP-GCs) and the DG (DG-GCs) of wild-type mice. Subsequent mass spectrometry and proteomic analysis revealed specific developmental trajectories for the different subregions and highlighted distinct functional states of HP-GCs and DG-GCs.

## RESULTS

### Characterization of the critical time window for early postnatal development in the hippocampus

**Fig. 1.**
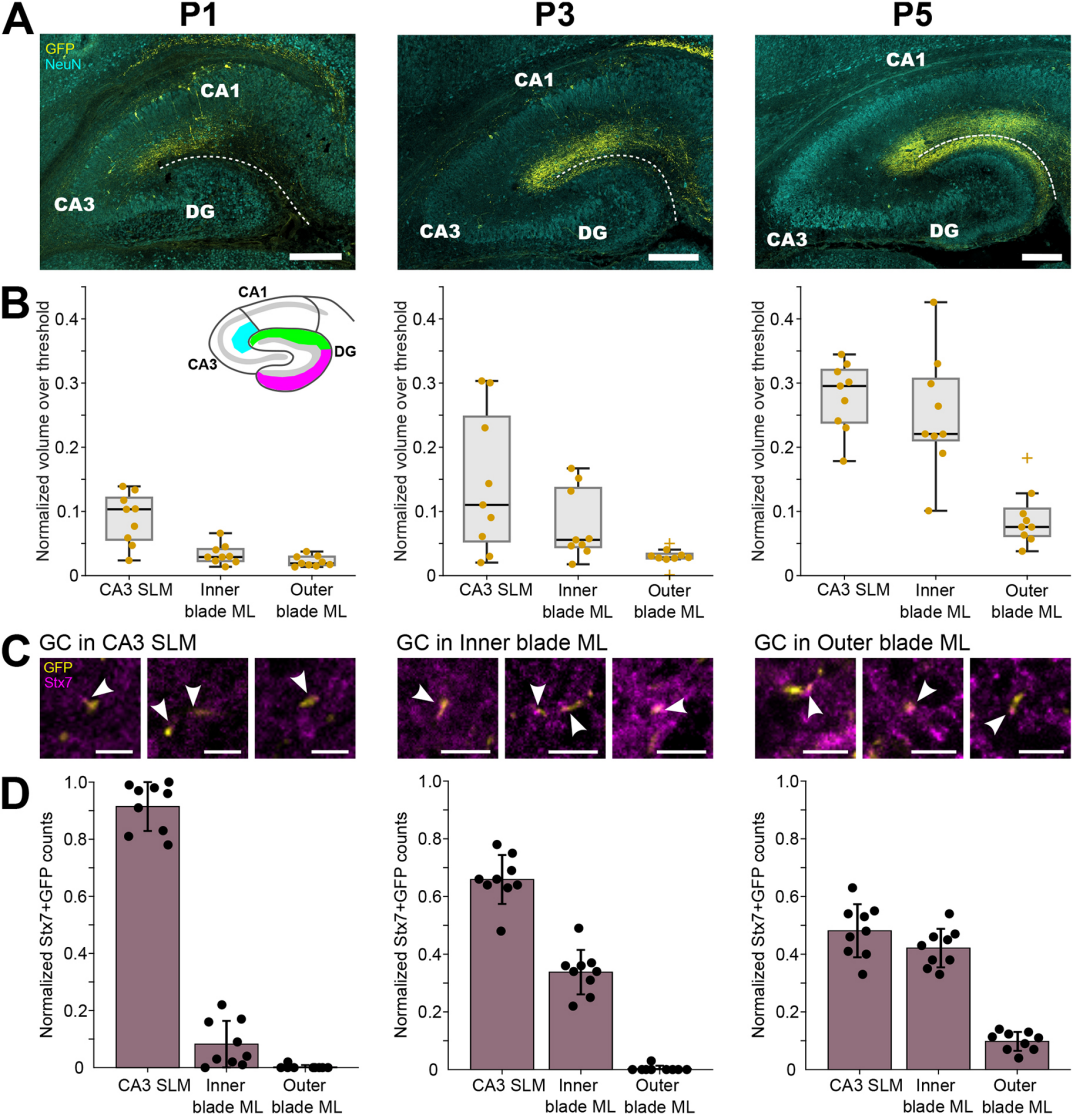
**Invasion of the hippocampus by the fibres of entorhinal cortex reelin-positive Layer 2 cells.** (A) Confocal image of horizontal sections of the hippocampus (HP) of Odz3-tTa;TetO-GCamP6 animals at P1, P3 and P5. NeuN staining (turquoise) is used to label neuronal cell bodies. Staining of the GFP part of GCamP6 (GFP) labels fibres of reelin-positive Layer 2 cells (yellow). Dashed line indicates the hippocampal fissure. (B) Quantification of the increase in GFP fluorescent signal in the stratum lacunosum-moleculare of cornu ammonis 3 (CA3 SLM) and in the molecular layer (ML) of the inner and outer blade of the dentate gyrus (DG) over time reflecting how the invasion progresses during the early postnatal days. Depicted is the normalized volume of fluorescent voxels over the background threshold for each subregion of interest. Datapoints identified as outliers (quartile method) are depicted as crosses. Inlay shows schematic of quantified areas (CA3 SLM in turquoise, inner blade DG in green, outer blade DG in pink) and a represented delineated image for quantification is shown in Fig. S1B. (C) Representative confocal images of growth cones (GCs) of reelin-positive Layer 2 cells in CA3 SLM at P1 (left), inner ML at P3 (middle) and outer ML at P5 (right). GCs are indicated by Stx7 puncta (magenta) at the end of GFP-positive fibres (yellow); see arrows. (D) Quantification of GC density. Bar plots show normalized mean and standard deviation of Stx7+GFP-positive events for each delineated area. Three non-consecutive slices of three different animals were counted for the Stx7+GFP events for each timepoint (*n*=9). Given numbers are normalized to total counts per slice. Scale bars: 200 μm (A); 5 μm (C).

Hippocampal subregion connectivity is established during early postnatal development (Donato et al., 2017; Dumas, 2005). A key event is the invasion of the HP and its subregions by its major input, the reelin-positive Layer 2 cells of the EC. An early study identified P3-P6 as the critical window in rat DG invasion, though it was limited in its timeline and precision in labelling of the invading fibres (Fricke and Cowan, 1977). To better characterize the ingrowth dynamics of reelin-positive Layer 2 fibres in mice, we used a recently developed transgenic mouse line which specifically labels those cells. The mouse line conditionally expresses the tetracycline-controlled activator protein (tTa) under the control of the reelin-positive Layer 2 cell-specific enhancer element Odz3 (in the following named Odz3-tTa) (Blankvoort et al., 2018). To label the axons of interest, we crossed this line with a TetO-GCamP6 reporter mouse line (Blankvoort et al., 2018) and performed immunohistochemical staining for the GFP part of GCamP6 at P1, P3 and P5 (Fig. 1A; Fig. S1A). Our analysis showed that input fibres are present in the CA regions of the HP at P1, reaching CA3. At P3, we observed fibres in the inner blade of the DG, starting to invade the outer blade at P5 (Fig. 1A). The increase in numbers of invading fibres corresponded with an intensification of the fluorescent signal in the different hippocampal subregions (Fig. 1B; Fig. S1B). To confirm that the increase in fluorescent signal reflected new axon invasion to the specific subregions, we co-labelled for the GC marker for Syntaxin7 (Stx7) (Nozumi et al., 2009). Colocalization of GFP and Stx7 signals allowed us to identify GCs of axons from EC reelin-positive Layer 2 fibres (Fig. 1C). Across nine sections from three animals per timepoint, we counted a total of 1734 (P1), 1970 (P3) and 8256 (P5) GCs, demonstrating a substantial increase in arriving EC fibres (Fig. S1C). Indeed, at P1, most GCs localized to CA3 SLM near to the DG inner blade border, with progressive increases in the inner (P3) and outer (P5) blade of the DG (Fig. 1D).

These findings demonstrate a clear sequential invasion of HP subregions by EC reelin-positive Layer 2 axons, with the DG being the final target and P3-P5 representing a particularly critical time window for the development of the connectivity in the DG. We therefore selected these three timepoints for proteomic GC analysis.

### Quality control experiments for growth cone extraction of microdissected HP and DG

**Fig. 2.**
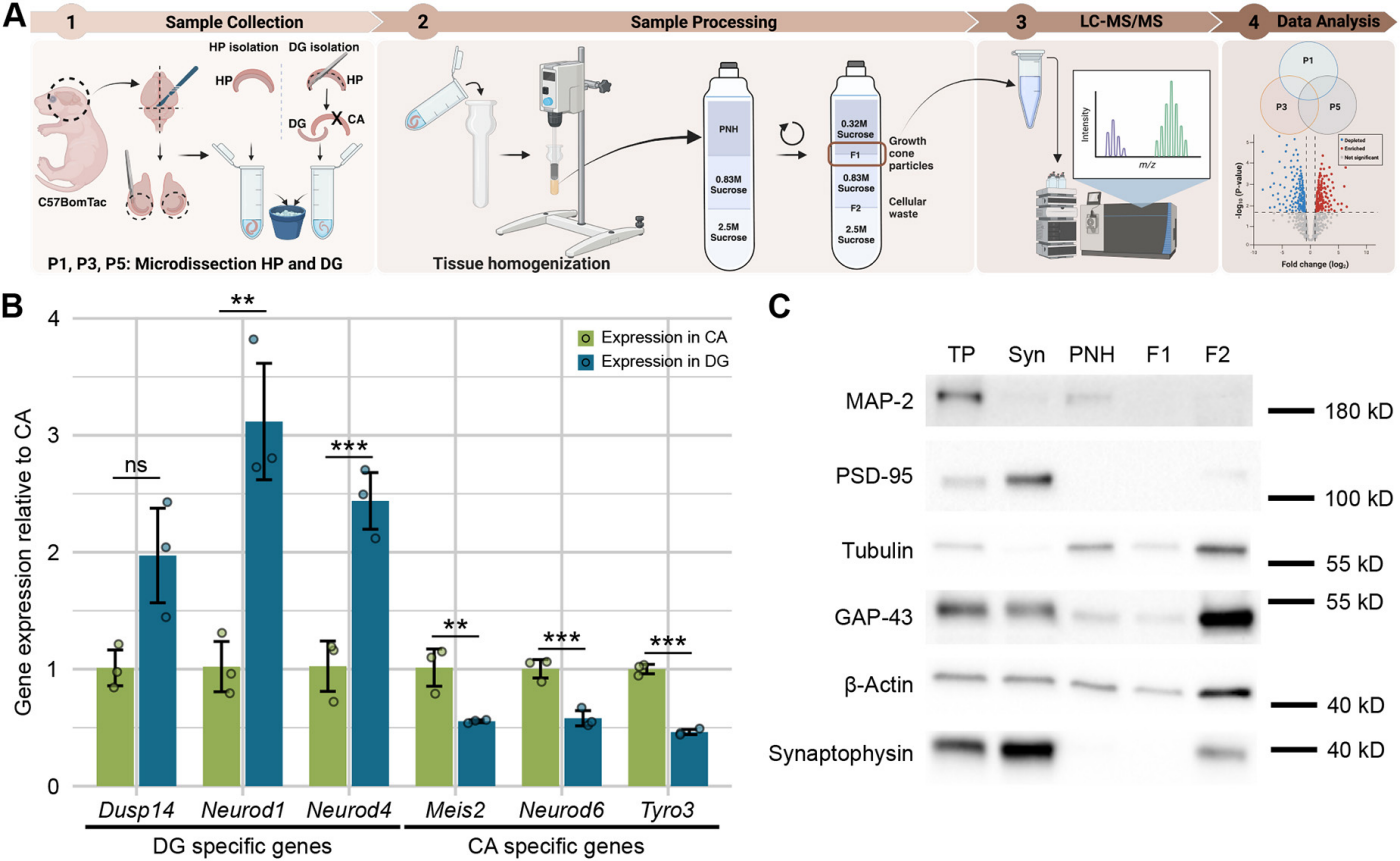
**Validation of growth cone isolation protocol.** (A) Overview for the sample preparation. Created in BioRender by Quattrocolo, G., 2026. https://BioRender.com/itle6j1. This figure was sublicensed under CC-BY 4.0 terms. (B) qRT-PCR results to verify the precision of dentate gyrus (DG) dissection in P4 wild-type animals. Depicted is the relative gene expression to the cornu ammonis (CA) tissue for DG-associated genes (*Neurod1*, *Neurod4* and *Dusp14*) and CA-associated genes (*Meis2*, *Neurod6* and *Tyro3*). Significance was tested using an independent two-tailed Student's *t*-test and significance levels are indicated as **P<0.01 and ***P<0.005. ns, non-significant. *n*=3. (C) Western blot analysis as quality control for growth cone (GC) preparation of P4 wild-type animals. Post nuclear homogenate (PNH) and the two fractions after density gradient centrifugation F1 (washed) and F2 of the GC preparation process were analysed for different GC and maturity markers. Total adult hippocampal protein extract (TP) and adult synaptosome preparations (Syn), both P80, serve as positive controls for antibodies against maturity markers. We loaded 1 μg of protein per sample. The neuronal marker MAP-2, the pre- and postsynaptic markers synaptophysin and PSD-95, respectively, to exclude mature structures in our sample preparation, and the cytoskeleton components tubulin and β-actin were detected, as well as the GC marker GAP-43 to show the presence of GCs.

To study all GCs during early postnatal development of the HP on a proteomic level, we aimed to extract GCs from the total HP and the DG subregion of wild-type mice to submit them to mass spectrometry (Fig. 2A). Before this, we performed a series of control experiments to assess different steps of our isolation protocol.

First, the precision of tissue dissection was verified with qRT-PCR at P4 using region-specific genes identified from the Allen Developing Mouse Brain Atlas (https://developingmouse.brain-map.org/) and known adult markers: DG- and CA-specific target genes were *Neurod1*, *Neurod4*, *Dusp14* and *Meis2*, *Neurod6*, *Tyro3*, respectively. DG-associated genes showed a significantly higher mRNA level for DG tissue, while CA-associated genes were clearly more abundant in the CA tissue extracts, confirming our ability to correctly dissect the DG from the HP even at these early developmental stages (Fig. 2B).

GCs from total HP and the DG were isolated using a modified protocol (Lohse et al., 1996). After sucrose density gradient separation, GCs were collected from the F1 fraction, while the remaining post nuclear homogenate (PNH) components were collected in the F2 fraction of the gradient. F1 fractions were washed with PBS to reduce potential protein contamination (Pfenninger et al., 1983) and increase the amount of GCs compared to unwashed GC preparations (Fig. S2A). Western blot validation of total HP-GC preparation at P4 (Fig. 2C; Fig. S2B), with adult hippocampal total protein (TP) and synaptosome (Syn) preparations as controls for antibody functionality, showed expected results. PSD-95 and synaptophysin were detectable only in adult controls and MAP-2 was mainly present in mature tissue with faint signal in the PNH sample but absent from GC fractions. Cytoskeletal protein tubulin and β-actin were detected in nearly all fractions except tubulin in synaptosomes. GAP-43, a classical GC marker, was detected across all fractions, consistent with its broad expression throughout developing neurons, explaining the strong F2 fraction signal.

Summarizing, we were able to detect all GC-associated proteins (GAP-43, tubulin and β-actin) in the F1 fraction. Collectively, our control experiments confirmed the validity of our preparation of GCs from microdissected DG and HP (Fig. 2).

### Hippocampal growth cones in early postnatal days

With these quality controls in place, we characterized the proteomic landscape of HP-GCs. We sampled five animals individually for each timepoint P1, P3 and P5 by pooling their HPs and isolating the GCs (five biological replicates per timepoint). Extracted GCs were filtered on 0.1 μm filters and washed extensively to remove protein contaminations before liquid chromatography-tandem mass spectrometry (LC-MS/MS) analysis. Samples were measured in two separate batches of three and two biological replicates. Raw data were processed using MaxQuant v.2.6.1 (Cox and Mann, 2008) and subsequent data analyses were conducted with log2 transformed label free quantification (LFQ) intensities with R. Proteins were counted as identified when detected in three out of five replicates. Comparison of the log2 LFQ intensities for known GC markers in our dataset between batches showed no differences, confirming no batch effect.

**Fig. 3.**
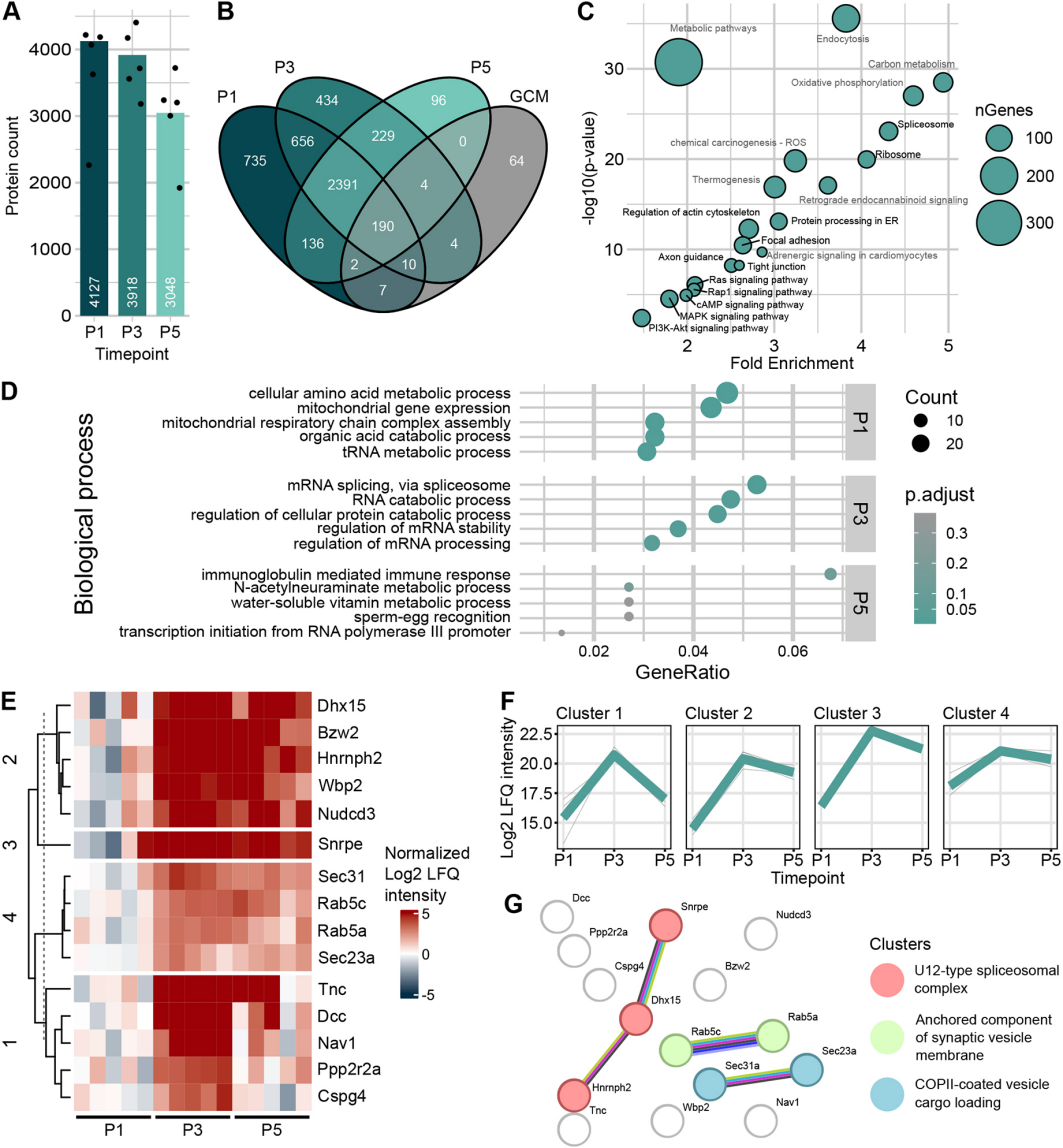
**Proteome of hippocampal growth cones.** (A) Bar graph representing the number of identified proteins per timepoint (*n*=5 animals for each timepoint). (B) Venn diagram showing the distribution and overlap of identified proteins for each timepoint, and the overlap with a list of growth cone markers (GCM) from Chauhan et al. (2020). (C) KEGG pathways identified for the proteome shared across timepoints. Relevant growth cone (GC) pathway terms are highlighted in black. (D) List of Top 5 Gene Ontology terms for biological processes for the timepoint-specific proteins at P1, P3 and P5. Dot size indicates the protein count per term and the dot colour indicates the adjusted *P*-value. (E) Heatmap representing expression across development normalized to the mean log2 LFQ intensity at P1 of proteins identified as significantly different in the maSigPro single time series analysis. Clusters were defined using the single time series regression analysis and k-means clustering. See also Table S1. (F) Detailed temporal trajectories for each cluster shown in E for the single time series temporal trajectory analysis. Average log2 LFQ intensities for all proteins in the cluster are represented in teal and the individual protein signals in grey. (G) STRING network analysis of all significant proteins of the temporal analyses with all known interactions between proteins, depicted in a default colour code. Three functional clusters were identified within the STRING network.

We identified 4894 proteins in total, averaging of 3500-4000 proteins per timepoint (Fig. 3A). Venn diagram analysis revealed timepoint-specific protein subsets (Fig. 3B) with P1 showing the largest (∼800 proteins) and P5 the smallest (∼100 proteins) subsets. Comparison with a comprehensive GC marker (GCM) list showed ∼80% on our dataset, consistent with published values (Chauhan et al., 2020). We further assessed the reliability of our GC dataset by analysing the KEGG pathways [false discovery rate (FDR) of 0.05] covered by the proteome shared by the three different timepoints (Fig. 3C). We identified expected GC-associated pathways among the Top 20 (disease terms excluded), including ‘Regulation of actin cytoskeleton’, ‘Focal adhesion’, ‘Axon guidance’, ‘Ras signalling pathway’ and ‘Rap1 signalling pathway’*.* Interestingly, ‘Ribosomes’ and ‘Spliceosomes’ were among the most enriched pathways. These molecular machineries are essential for local mRNA modification and translation, enabling the GCs to rapidly remodel their local proteome in response to dynamic guiding cues (Cagnetta et al., 2018; Shigeoka et al., 2016).

Gene ontology (GO) term analysis of timepoint-specific proteins for HP-GCs at P1, P3 and P5 revealed distinct biological signatures (Fig. 3D). At P1, the most abundant terms were associated with mitochondrial processes such as ‘Mitochondrial gene expression’ and ‘Mitochondrial respiratory chain complex assembly’, potentially reflecting the high energy demand in GCs for active axon extension, cytoskeletal remodelling and membrane synthesis by ensuring robust energy-producing machinery (Smith and Gallo, 2018; Vaarmann et al., 2016). Interestingly, ‘tRNA metabolic processes’ was also highly enriched, underlying the high demand for efficient local protein synthesis at early timepoints (Estrada-Bernal et al., 2012; Harnett et al., 2022).

Biological processes for P3-specific proteins were mainly associated with ‘RNA catabolic processes’ and ‘RNA splicing’. Active response to guiding cues and the establishment of synaptic contacts requires a rapid mRNA turnover (Shigeoka et al., 2016) and splicing would allow GCs to fine-tune responses by generating developmentally relevant isoforms of key proteins, such as cell adhesion molecules and guidance receptors (Chen et al., 2008). This would not only contribute to circuit formation (Shigeoka et al., 2016) but could also indicate molecular reprogramming as HP-GCs transition from pathfinding to stable connectivity. At P5, although GO terms showed overall lower significance, ‘Immunoglobulin mediated immune response’ stood out. This term is triggered among others by the protein C1qb, the core component of the complement C1 complex. Beyond its immune function, this complex is crucial for microglia-mediated synaptic pruning, a process with immune-like mechanisms that eliminate excess synaptic connections in GCs and developing circuits (Stevens et al., 2007). The appearance of these specific terms only at P5 could further suggests a functional transition of HP-GCs from the exploration phase to the formation of stable connections.

To evaluate temporal dynamics in the HP-GC proteome, we conducted a single-time series analysis using the maSigPro R package (https://bioconductor.org/packages/maSigPro) including all proteins, using P1 as reference timepoint and setting the minimum observations to four. After least square linear regression of the log2 LFQ intensities and a Benjamini–Hochberg correction (*P*<0.05), 15 proteins showed significant changes across timepoints (Fig. 3E; Table S1). K-means clustering represented the individual changes best with four clusters, showing a similar pattern of increase from P1 to P3, followed by a decline toward P5 (Fig. 3F). Indeed, nearly all significant proteins identified in the single-time series analysis peaked around P3, indicating this timepoint as critical in the HP-GC maturation.

To highlight possible interactions, we performed STRING network analysis for the fifteen significant proteins together, revealing three functional networks (Fig. 3G). The first included the ‘U12-type spliceosome complex’, which processes a small subset of introns in genes involved in cell cycle regulation and development (Edery et al., 2011; Otake et al., 2002). Interestingly, this network also includes the heterogeneous nuclear ribonucleoprotein H2 (HNRNPH2), a protein with a nuclear localization sequence (NLS) (Gonzalez et al., 2023) and regulatory effect on alternative splicing (Rappe et al., 2014), suggesting communication between GCs and the nucleus. The second network, ‘Anchored components of synaptic vesicle membrane’, further highlights the transition to active synaptogenesis and synaptic maturation. The third, ‘COPII-coated vesicle cargo loading’, indicates increased endoplasmic reticulum (ER)-to-Golgi protein trafficking to the GCs, notably including the GC marker SEC31A (outer shell component of COPII) (Chauhan et al., 2020). Among the 15 temporal changing proteins, but outside any network, was DCC (DCC netrin 1 receptor), a protein well characterized for mediating axon attraction upon ligand binding (Powell et al., 2008; Ren et al., 2007) and known to regulate local axonal translation through ribosome binding (Koppers et al., 2019).

Together, these observations indicate a coordinated temporal regulation of mitochondrial activity, RNA processing and protein trafficking during the early postnatal HP-GC development. The maSigPro analysis highlights P3 as a potential critical timepoint, marking the transition from axonal growth and exploration to synaptic maturation.

### Growth cones of the DG in early postnatal days

**Fig. 4.**
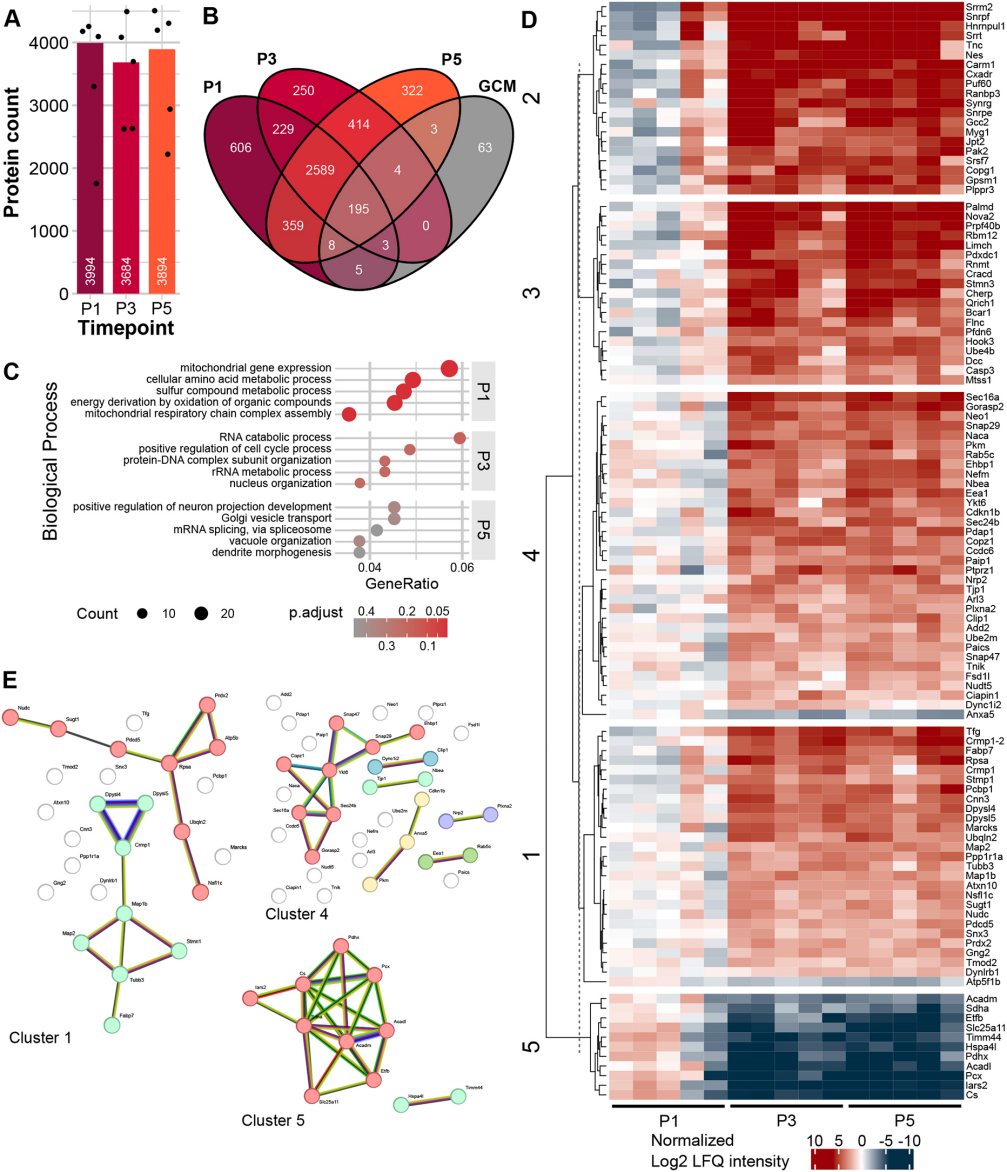
**Proteome of dentate gyrus growth cones.** (A) Bar graph representing the number of identified proteins per timepoint (*n*=5 animals for each timepoint). (B) Venn diagram showing the distribution and overlap of proteins identified for each timepoint and the overlap with the growth cone (GC) marker reference dataset of Chauhan et al. (2020). (C) List of Top 5 results for the Gene Ontology term analysis for timepoint-specific proteins in the dentate gyrus growth cone (DG-GC) dataset. (D) Heatmap showing the normalized log2 LFQ intensity and results of k-means clustering – 111 significantly changed proteins are represented. Protein names and their clusters can be found in Table S1. (E) Results for functional network analysis for clusters 1, 4 and 5 conducted with STRING network analysis.

Knowing that the DG is the last subregion in the HP receiving its input from the Layer 2 cells of the EC, we then asked whether GCs of the DG show a distinct proteomic profile. As for the HP-GCs, we sampled wild-type pups at P1, P3 and P5, pooling both DG of one animal as one biological replicate. Five biological replicates were generated for each timepoint and analysed in two batches, as previously described for HP-GCs. We identified ∼4000 proteins for the DG-GC samples from P1, P3 and P5 (Fig. 4A), and a total of 4987 proteins overall. As in the HP-GCs dataset, P1 showed the highest number of timepoint-specific proteins. However, in DG-GCs, the lowest number was observed at P3, while P5 showed a three times higher number compared to HP-GCs, suggesting ongoing dynamic proteomic changes. The Venn diagram for DG-GCs (Fig. 4B) additionally revealed that the P3 and P5 samples showed a larger overlap in their proteome than the corresponding HP-GC samples (DG-GCs 414 proteins versus HP-GCs 239 proteins), suggesting that P3 and P5 DG-GCs might be more similar than the corresponding samples in HP-GCs. Also in this case, the comparison to the GC markers resulted in ∼80% overlap, with similar results to what we saw for the HP-GCs. KEGG pathway analysis for the proteome shared by the three different timepoints revealed GC-associated pathways and resulted in the same Top 20 terms that were identified for the HP-GCs shared proteome, except for two terms ‘Chemokine signalling pathway’ and ‘Adrenergic signalling in cardiomyocytes’ (Fig. S3).

To achieve a deeper insight into the functions of the identified proteins, we again ran a GO term analysis for the timepoint-specific proteins of DG-GCs (P1: 606; P3: 250; P5: 322) (Fig. 4C). At P1, we observed similar terms to what was previously discussed for the HP-GCs, with biological processes associated with ‘Mitochondrial gene expression’ and ‘Respiratory chain complex assembly’, as well as tRNA metabolic processes, underscoring that GCs at P1 are in a state of high energy demand and active protein synthesis. While P3 and P5 GO terms showed lower significance, the majority differed from the HP-GC terms. At P3, among the highest rated terms were ‘RNA catabolic processes’ and ‘Protein-DNA complex subunit organization’. Terms related to RNA metabolism were common between the two HP-GC- and DG-GC-P3-specific GO terms, suggesting that P3 is very dynamic in what relates to RNA transcription. The term ‘Protein-DNA complex subunit organization’ was triggered by members of the histone family (H2BC4) and their potential regulatory interaction partner (SET, ANP32E, ANP32B, TAF10). Histones have been observed in cerebellar neurites (Mishra et al., 2010) as well as in the axonal proteome of developing isolated retinal axons (Cagnetta et al., 2018; Moretti et al., 2015). The ANP32 protein family covers diverse functions, from chromatin regulation to intracellular transport, and is important for normal development (Reilly et al., 2011). As previously mentioned, in the P3 HP-GCs, the major biological processes were related to local mRNA alteration via splicing. The splicing term was also detected for DG-GCs, however much less abundant (count) and less significant (adjusted *P*-value) and at a later timepoint (P5, Fig. 4C). Besides, the analysis of GO terms of DG-GCs at P5 highlighted two top terms, ‘Positive regulation of neuron projection development’ and ‘Golgi vesicle transport’, suggesting that at P5 GCs from the DG are still in a process of dynamic exploration.

Summarizing, these observations on timepoint-specific proteins reflect a shift in the timescale of GC development in the DG: while HP-GCs start to transition from mobile exploring structures to local entities forming first stable connection between P3 and P5, GCs of the DG might still be in their exploration phase. This would reflect the timeline of invasion of the DG by its major input, the axons of entorhinal Layer 2 cells, that have not fully invaded the outer blade of the DG until P5 (Fig. 1A).

To highlight the trajectory of DG-GCs, we ran a single time series analysis also for DG-GCs, which resulted in 111 significantly differently expressed proteins across the three analysed timepoints (Fig. 4D; Table S1). The changes in Top 20 LFQ intensity were best represented in five clusters using k-means clustering (Fig. 4D). While clusters 1-4 exhibited increasing intensities over time, cluster 5 was the only cluster that represents proteins that are decreasing in intensity at the later timepoints. The most abundant proteins overall were found in cluster 1. In fact, clusters 2, 3 and 4 were primarily distinguished by their initial intensities at P1 and the following trajectory of increase (Fig. 4D; Fig. S4A).

To assess protein-protein interactions within each temporal profile, we performed STRING network analysis independently for each cluster (Fig. 4E; Fig. S4B,C). Two functional networks were identified in cluster 1. One network (in pale green, Fig. 4E) is associated with both the ‘CRMPs in Sema3A signalling’ and ‘Negative regulation of microtubule polymerization or depolymerization’. CRMPs are PlexinA-interacting proteins that act as mediator of SEMA3A signalling and neuronal differentiation (Schmidt and Strittmatter, 2007). Phosphorylation of CRMPs inhibits their ability to bind tubulin dimers, leading to destabilization of the microtubule and ultimately causing the collapse of the GC (Arimura et al., 2005). Also part of this network is the fatty acid binding protein-7 (FABP7), a brain-specific lipid chaperone that plays essential roles during the studied developmental window by regulating the intracellular trafficking and metabolism of fatty acids and facilitating their transport to specific cellular compartments (Young et al., 2013). The second network (red, Fig. 4E) includes proteins of mixed functionality, such as NUDC (involved in neuronal migration; Aumais et al., 2001), UBQL2 (also known as UBQLN2) (a key player in proteostasis; Lin et al., 2022), ATP5B (mitochondrial energy metabolism; Chinopoulos et al., 2011) and RPSA (a small ribosomal subunit protein that acts as a laminin receptor involved in cell adhesion; Blazejewski et al., 2022).

Cluster 4 contained a total of six functional networks, with one major functional network (red, Fig. 4E) that represents the ‘SNARE complex and Snap receptor activity’. The SNARE (N-ethylmaleimide-sensitive factor attachment protein receptor) complex is mostly known for its involvement in membrane fusion and the synaptic vesicle cycle. Fusion of membrane vesicles managed by the SNAREs, with coordinated elongation of the cytoskeleton, is responsible for the growth of axons (Ulloa et al., 2018). Another important STRING network in cluster 4 represents the semaphorin-plexin signalling pathway (purple, Fig. 4E) involved in neuronal projection guidance with the proteins neuropilin 2 (NRP2) and plexin A2 (PLXNA2). Both proteins are known to be crucial for the development of the hippocampal mossy fibres and DG (Skutella and Nitsch, 2001; Zhao et al., 2018). The remaining cluster 4 networks are associated with retrograde microtubule transport, cell polarity and cell adhesion.

As mentioned above, cluster 5 is the only cluster that showed a decrease in log2 LFQ intensity in the single-time series analysis. As most of cluster 5 proteins (red, Fig. 4E) are associated with ‘Fatty acid beta-oxidation using acyl-CoA dehydrogenase’ and the ‘Citrate cycle’, this cluster represents energy production. The remaining two proteins (pale green, Fig. 4E), HSPA4L and TIMM44, are both involved in mitochondrial protein import (Ting et al., 2017). Thus, cluster 5 emphasizes a trend observed in the GO term analysis for the timepoint-specific proteins, as at P1 protein synthesis and energy production were among the most important terms, disappearing by P3 and indicating a shift in functional needs of the GCs (Fig. 4C).

Finally, clusters 2 and 3 contain one or two functional networks, respectively (Fig. S4B,C). Cluster 2 network proteins are associated with ‘RNA processing’ (red) and especially ‘mRNA splicing via spliceosome’ (blue) (Fig. S4B). Two STRING networks of cluster 3 represent the pathway ‘Caspase activation via dependence receptors in the absence of ligand’ and a second network without specific function (proteins: BCAR1 and FLNC) (Fig. S4C). Remarkably, most proteins of this cluster are labelled as phosphoproteins, including the guidance receptor DCC, cytoskeleton-interacting proteins (STMN3, FLNC, LIMCH1, BCAR1, MTSS1), and the protein degradation-associated ubiquitin factor E4B (UBE4B), and caspase 3 (CASP3). Phosphorylation of these proteins would allow for rapid and reversible modulation of protein localization, cytoskeletal dynamics and enzymatic activity, contributing to the synaptic characteristics of GCs during axon guidance and circuit formation (O'Donnell et al., 2009).

Together, the single-time series analysis of DG-GCs revealed a developmental trajectory characterized by increasing intensities across most clusters, contrasting the peak-and-decline pattern observed in HP-GCs. Identified functional networks of significantly changing proteins span key processes from cytoskeletal regulation, semaphorin signalling to SNARE-mediated membrane dynamics and energy metabolism, reflecting the diverse molecular demands of actively navigating GCs. The decrease of metabolic proteins (cluster 5) alongside the sustained increase of guidance and signalling components further supports the assumption that DG-GCs at P5 remain in an exploratory phase, consistent with the delayed arrival of EC input fibres in this subregion.

### Subregion specificity of HP-GCs and DG-GCs

The analysis of GC proteomes from the HP and DG showed that each timepoint is characterized by distinct protein subsets. P1 GCs of HP and DG share similar functional profiles, but from P3 onward the timelines diverge, indicating that the HP-GCs mature on average earlier than the DG-GCs. The averaged expression profiles of the different clusters identified in the time course analysis also differ, with the HP-GCs clusters having a clear reduction from P3 to P5 (Fig. 3F), in contrast to a much higher similarity in the DG-GCs clusters (Fig. S4). Therefore, to further understand the subregional differences in the two types of GCs, we did a comparative analysis of the proteomes generated for HP-GCs and DG-GCs.

**Fig. 5.**
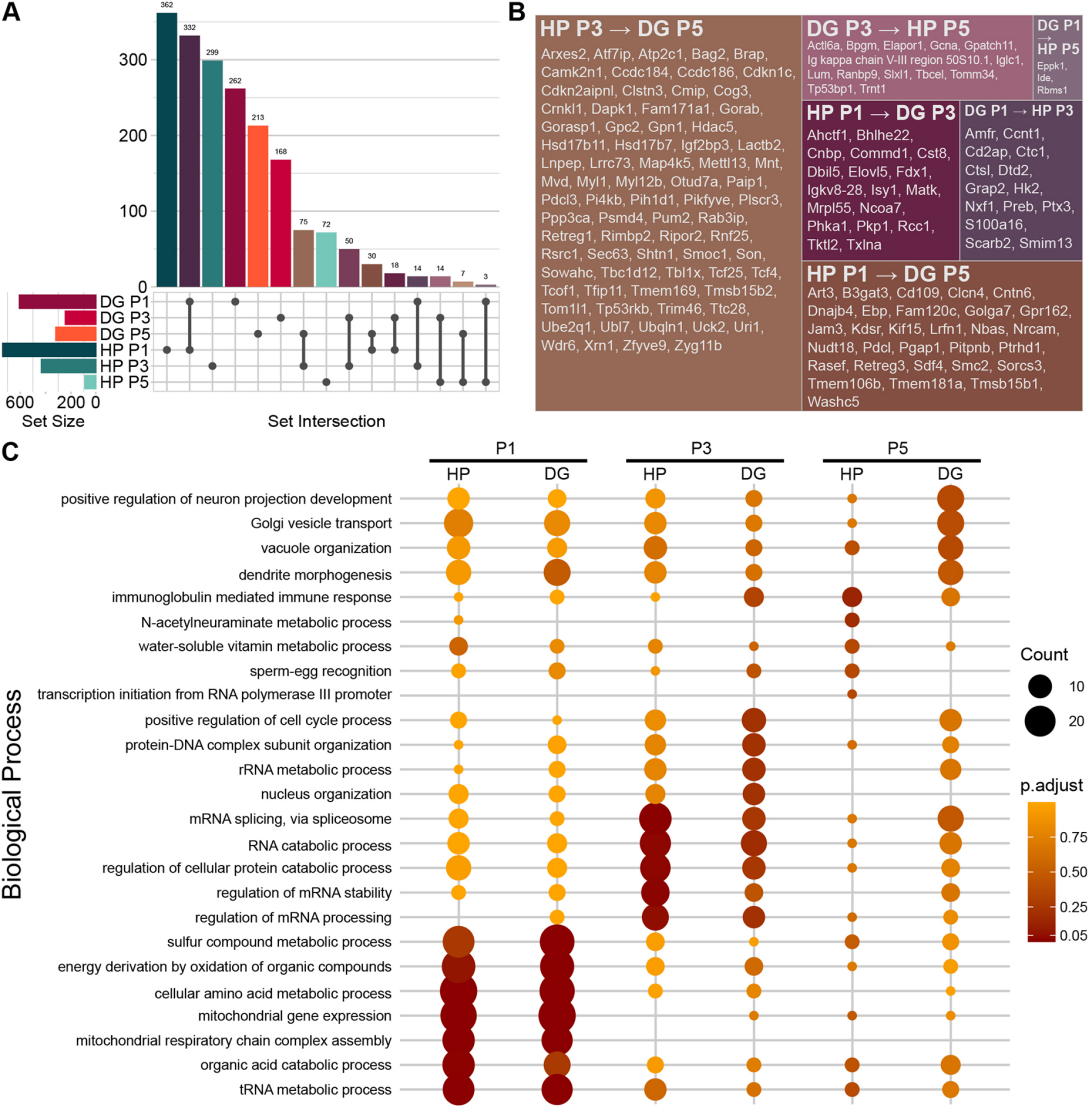
**Comparative analysis of time-specific proteins from hippocampal growth cones and dentate gyrus growth cones.** (A) Upset plot of the timepoint-specific proteins for hippocampal growth cones (HP-GCs) and dentate gyrus growth cones (DG-GCs). The lower left part of the plot represents the total number of proteins identified for P1, P3 and P5 in HP-GCs and DG-GCs. The lower right part represents the set intersection. Each set contributing to the intersection is marked with a point and connected with a line. The upper part of the plot gives the number that belongs to the set intersection indicated below. (B) Tile plot showing overlapping proteins between HP and DG at different timepoints. Colour code based on A. (C) Comparative depiction of the Top 5 biological processes of HP-GCs and DG-GCs from Figs 3D and 4C.

First, we analysed the overlap between the timepoint-specific proteins between the two datasets (Figs 3B and 4B). The Upset plot (Fig. 5A) shows the overlap between the six groups of timepoint-specific proteins. At P1, ∼50% of the time-specific proteins are shared between the HP and DG, explaining the similar biological processes in the GO term analysis. With increasing age, the overlap decreases from over 300 proteins at P1, to 50 at P3 and 7 at P5, indicating that HP-GCs and DG-GCs diverge over time. The overlap between P1 HP-GCs with P3 and P5 DG-GCs (30 and 18 proteins) and between P3 HP-GCs with P5 DG-GCs (75 proteins) supports a shifted maturation trajectory of the DG to a later timepoint as the HP (Fig. 5B). In the group of proteins shifted to DG P5 we observed several members of the transmembrane (TMEM) protein family. TMEMs are associated with various functions, such as maintaining cell homeostasis and cell-cell recognition (Chen et al., 2021; Herrera-Quiterio and Encarnación-Guevara, 2023). TMEM106B, for example, is involved in protein degradation processes via lysosomes and a recent study demonstrated that a TMEM106B variant promotes neurite outgrowth and later spine formation in hippocampal neurons *in vitro* (Nguyen et al., 2023). Also TRIM46 was found at P5 in the DG and is crucial for axon formation by regulating the microtubule organization (Harterink et al., 2019). This supports the idea that the GCs isolated at P5 from the DG are still in their elongating exploratory phase. Other interesting proteins found at later timepoints in the DG are PUM2, an RNA binding protein and master regulator of local translation (Hafner et al., 2010; Martínez et al., 2019), TMSB15B1 and TMSB15B2, both with assumed roles in cytoskeleton organization (Uniprot based on similarity), TOM1L1, which regulates endosomal trafficking and is important for growth factor signalling (Wang et al., 2010), and COMMD1, associated with endocytic recycling and protein trafficking (Phillips-Krawczak et al., 2015). Since these functionally important GC proteins were detected earlier in HP-GCs than DG-GCs, this further emphasized the temporal offset between the two GC populations. A small number of proteins detected earlier in DG-GCs than HP-GCs also highlights bidirectional developmental processes within the HP, such as putatively mossy fibres.

The temporal shift between the HP-GCs and DG-GCs is additionally highlighted when the Top 5 biological processes for each timepoint are plotted together (Fig. 5C). At P1 both share the same biological process, but from P3 onward the terms diverge, with splicing-associated terms persisting until P5 in DG-GCs while HP-GCs shifted to target recognition.

**Fig. 6.**
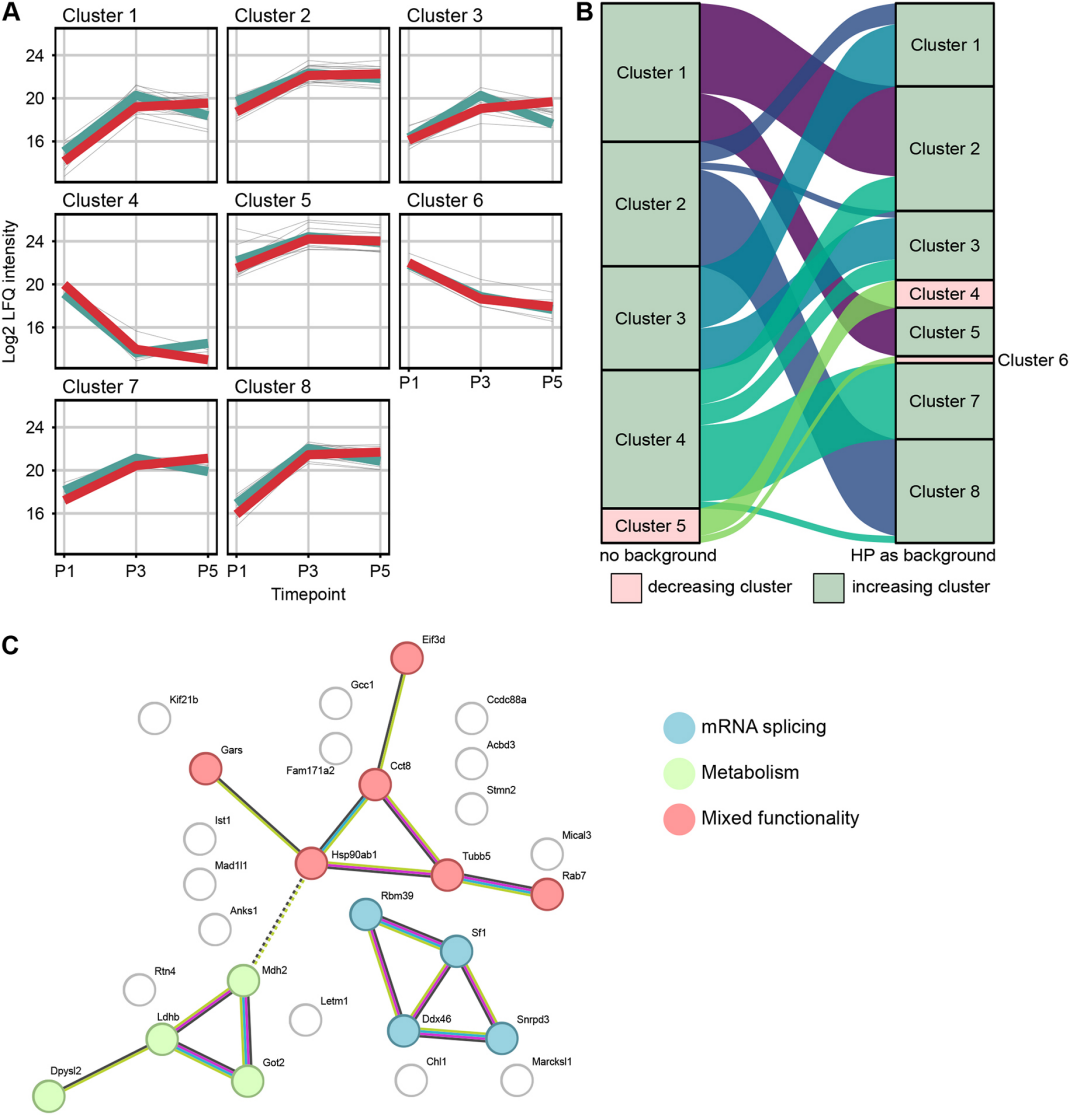
**Results of a multi-time series analysis with hippocampus as control.** (A) Average log2 LFQ intensities over time, with the average for hippocampal growth cones (HP-GCs) in teal and the average for dentate gyrus growth cones (DG-GCs) in red. Individual protein curves are in grey. The corresponding heatmap with normalized log2 LFQ intensities can be found in Fig. S5. A list of individual protein names and clusters can be found in Table S1. (B) Alluvial plot on clustering consistency between the single time series and multi time series analysis of shared proteins. Clusters with decreasing intensities are coloured in pink and clusters with increased intensities in green. (C) Results for STRING network analysis of proteins only identified as significantly changed in the multi-time series analysis.

Multi-time series analysis for DG-GCs against the HP-GCs enabled us to further identify tissue-specific developmental changes in LFQ intensity. The analysis was conducted on a non-imputed dataset, avoiding any possible assumption about the reason behind the missing proteins. Normalization to P1 HP-GCs values revealed LFQ intensities for 110 proteins significantly changing (*P*<0.05) within the early postnatal days (Table S1). K-means clustering was best with eight clusters, with six clusters with increasing and two clusters with decreasing trajectories (Fig. S5). Clusters 1 and 8 represent proteins with the largest increase between P1 and P5. Smaller increases are represented in clusters 2, 5 and 7, and moderate increases are represented in cluster 3 (Fig. 6A). HP-GCs show peak intensities at P3 in multiple clusters (1,3,4,7 and 8), while no DG-GC cluster shows a clear P3 peak (Fig. S4A). Decreasing intensities were found in clusters 4 and 6.

To evaluate if identical proteins were identified in the single- and multi-time series analysis, we compared the list of significantly temporally regulated proteins. We found 28 proteins unique to the multi-time series analysis, four shared with the HP single-time series, 74 shared with the DG single-time series, and four shared with both. The high overlap with the single-time series analysis of DG-GCs was expected, as only a few temporally regulated proteins were identified for the HP-GCs alone. To ensure the consistency in protein trajectory, we visualized the clusters in an alluvial plot and confirmed that no protein moved between increasing and decreasing clusters between the two analyses (Fig. 6B).

To evaluate the interaction for the 28 proteins additionally identified in the multi-time series analysis, we ran a STRING network analysis that resulted in three distinct functional networks (Fig. 6C) that are associated with mRNA splicing, metabolism, protein folding/stability [HSP90AB1 (Haase and Fitze, 2016) and CCT8 (Kelly et al., 2022)] and neuronal differentiation and dendritic spine formation [TUBB5 (Ngo et al., 2014) and RAB7 (Shikanai et al., 2018)]. The only proteins that did not show an increase in intensity in the multi-time series analysis were GOT2, LDHB, MDH2 (all metabolic) and LETM1 (mitochondrial morphology maintenance) (Jiang et al., 2013). In the metabolism network, the GC marker DPYSL2 (also known as CRMP2), was increased, which can be linked to its function in axon growth and guidance (Marques et al., 2013). Furthermore, DPYSL2 contains an NLS that could be indicative that DPYSL2, or one of its isoforms (Feuer et al., 2023), has another function in GC-nucleus communication.

The comparative analysis of HP-GCs and DG-GCs revealed subregion-specific developmental programmes. While most temporally regulated proteins in HP-GCs showed peak intensity at ∼P3 (Fig. 6A, clusters 1, 3, 4, 7 and 8), DG-GC proteins exhibited more sustained intensity without a clear P3 peak. Remarkably, six out of eight clusters in the multi-time series analysis showed increasing trajectory, which reflects intensive biosynthetic activity characteristic for critical developmental windows. The decrease of metabolic proteins from P1 to P5 may indicate a developmental shift from more general metabolic support to more specialized neuronal functions as proteins associated with metabolic processes decrease in their intensities while, at the same time, those important for modifying local translation and protein stability increase. This indicates a shift from an energy-producing and consuming phase to one in need of a new subset of proteins and function.

In summary, the comparative analysis of HP-GCs and DG-GCs confirmed the presence of subregion-specific developmental programmes with distinct temporal dynamics. These findings reinforce the view that the DG undergoes a shifted developmental timeline relative to the HP.

### Time-regulated proteins associated with neurological disorders

Beyond their developmental roles, many of the temporally regulated proteins in our dataset, including CASP3 and DPYSL2, are also implied in neurological diseases. CASP3 governs various roles in several diseases, among which are Alzheimer's disease, Amyotrophic lateral sclerosis (ALS), autism spectrum disorders and epilepsy syndrome (McIlwain et al., 2013). The loss of function of DPYSL2 has also been linked to epilepsy syndrome (Khanna et al., 2012). We used disease ontology (DO) analysis to cross reference the time-regulated proteins in our GC datasets with neurological disorders and neurodegenerative diseases (Table 1). Overall, around 20 of the time-regulated proteins were identified to be disease or disorder associated and are therefore of special interest, because these proteins might exhibit key functions during the development of the HP and DG. Disrupting their functions might lead to impairments in GC dynamics, cytoskeletal regulation and axonal guidance, potentially contributing to both neurodevelopmental disorders and, in the long term, to neurodegenerative diseases (Weerasinghe-Mudiyanselage et al., 2022). In addition to CASP3 and DPYSL2, RTN4 and RAB5A are also time-regulated in our dataset and have been associated with axonal local translation. Several studies have suggested that defects in the axonal mRNA translation may contribute to the progression of ALS, highlighting the importance of the RNA-associated proteins in our dataset (Cagnetta et al., 2018; Murakami et al., 2015). Table 1 provides a summary of all time-related proteins and their associated neurological disorders and neurodegenerative diseases. A comprehensive overview of all disease-associated proteins in the GC dataset is given in Table S2.

**Table 1.** Neurological and neurodegenerative diseases associated with time-regulated proteins in growth cone dataset (human orthologue genes)

Disease	Time-regulated orthologue genes
Alzheimer's disease	CDKN1B, RAB7A, STMN1, NACA, CASP3, RTN4, RAB5A
Amyotrophic lateral sclerosis (ALS)	CASP3, CSPG4, RTN4, RAB5A
Ataxia telangiectasia	CASP3
Autism spectrum disorder	NRP2, CASP3, NBEA
Charcot-Marie-Tooth disease	GARS1
Encephalitis	TJP1
Epilepsy syndrome	DPYSL2, CASP3, RTN4
Parkinson's disease	CASP3, PRDX2
Pick's disease	PRDX2
Prion disease	NES
Spinal muscular atrophy	GARS1
Spinocerebellar ataxia	ATXN10

The table depends on disease term enrichment analysis and selection of relevant diseases.

## DISCUSSION

The early postnatal period represents a critical developmental window for hippocampal circuit assembly. Our characterization of hippocampal invasion by EC Layer 2 reelin-expressing neurons confirmed that P1, P3 and P5 correspond to particularly dynamic phases of the entorhinal-hippocampal circuit development. At P1, the basic hippocampal architecture is established, though many connections remain immature with granule cell neurogenesis in the DG continuing at high rates and peaking at ∼P5-P7 (Bayer, 1980; Schlessinger et al., 1975). P3 represents a critical transitional period when EC projections undergo refinement (Amaral and Dent, 1981; Supèr and Soriano, 1994) and the laminar termination pattern become more defined (Amaral and Dent, 1981; Pleasure et al., 2000). By P5, coordinated network activity patterns emerge, which are thought to drive circuit maturation and functional development (Ben-Ari et al., 1989; Khazipov and Luhmann, 2006), representing a crucial time for experience-dependent plasticity mechanisms shaping final circuit organization.

Our proteomic analysis of HP-GCs and DG-GCs across these timepoints identified over 5000 proteins, with each timepoint characterized by a distinct protein subset (Figs 3B and 4B). Interestingly, timepoint-specific proteins covered very similar biological processes in HP and DG at P1, but with increasing age those processes diverge, demonstrating that the connections in DG develop on a slower timeline than the HP overall (Fig. 5C). This temporal offset was particularly evident for proteins involved in ‘RNA splicing via spliceosome’. Spliceosome-associated proteins appeared at earlier timepoints in HP-GCs but later in DG-GCs. The prominence of ‘Ribosomes’ and ‘Spliceosomes’ among the Top 20 KEGG pathways for shared proteins across all timepoints underscores two critical aspects of GC biology (Fig. S3). First, it highlights the importance of local protein synthesis for GC function. Second, it demonstrates the capacity for rapid proteome modulation through alternative splicing enabling fast responses to local environmental cues and guidance signals (Cagnetta et al., 2018; Estrada-Bernal et al., 2012; Shigeoka et al., 2016).

The overall shift in biological processes across developmental stages indicates that GCs mature from a mobile, exploratory state toward one focused on synaptic maturation and stable connection formation. P1 was primarily characterized by metabolic functions, particularly fatty acid metabolism. Among temporally regulated DG-GC proteins, we identified the lipid-interacting protein FABP7, which modulates fatty acid availability for membrane synthesis, indicating its involvement in coordinating lipid metabolism with neuronal maturation (Young et al., 2013). The importance of fatty acid metabolism for GCs was also demonstrated in previous studies (Chauhan et al., 2020). Given that FABP7 deficiency impairs hippocampal neurogenesis and alters behavioural outcomes (Young et al., 2013), disruptions in FABP7-mediated lipid metabolism during these critical early postnatal days could have lasting consequences for circuit development and function.

Our temporal trajectory analysis for HP-GCs revealed 15 significantly regulated proteins. While this number may seem low, it likely reflects the cellular heterogeneity within our samples, as the HP-GC preparation contained GCs from multiple cell types (EC projecting neurons, DG granular cell, CA neurons). The 15 temporally regulated proteins thus likely represent those with fundamental importance across HP subregions. Among these proteins, the GC marker DCC has been shown to function in axon guidance (Powell et al., 2008; Ren et al., 2007). In the context of high translational activity observed in our dataset, the ability of DCC to bind and regulate local translation is especially significant (Koppers et al., 2019; Koppers and Holt, 2022). Importantly, DCC was also identified as temporally regulated in DG-GCs, indicating its general importance across all HP subregions.

Interestingly, we identified several temporally regulated proteins that have been associated with transcription regulation, such as DPYSL2, HNRNPH2, FABP7 and MEF2C. DPYSL2, with functions ranging from axon guidance and cytoskeletal interactions to endocytosis and neurotransmission (Feuer et al., 2023), has been suggested to participate in nuclear signalling (Lu et al., 2021). HNRNPH2 is involved in RNA processing, alternative splicing, and mRNA trafficking, and shuttles between nucleus and cytoplasm to access pre-mRNA (Korff et al., 2023), while FABP7 mediates lipid-dependent transcriptional regulation after binding fatty acids (Needham et al., 2022). The presence of these proteins in GCs suggests a dual functional capacity, executing local functions while simultaneously communicating extracellular cues to the nucleus for transcriptional adaptation. This concept is well illustrated by transcription factor MEF2C, knockdown of which in postnatal neurons increased expression of the guidance cue ephrin A5, resulting in reduced collateral targeting (Sudarsanam et al., 2025). This highlights the direct interplay between nuclear transcription and axonal guidance. We identified MEF2C as temporally regulated in DG-GCs, and it will be fascinating to investigate the role of this transcription factor in GCs.

Furthermore, our dataset contains over 400 proteins associated with neurological and neurodegenerative diseases, with 20 showing temporal regulation. Disruptions in GC function have been implicated in various neurodevelopmental disorders, including autism spectrum disorders, ALS and epilepsy. This knowledge is particularly relevant given the growing recognition that many neurological and psychiatric disorders have their origins in developmental disruptions that occur during critical periods of circuit formation (Parenti et al., 2020).

Our study has limitations that should be considered when interpreting our findings. Our proteomic samples contained GCs from multiple cell types and, consequently, we cannot definitively attribute individual proteins and their functions to specific cell types within the HP. While comparing HP-GCs with DG-GCs allows us to speculate about DG-specific proteins, we cannot determine whether these proteins function primarily in extending mossy fibres or invading Layer 2 fibres. More refined extraction approaches, e.g. based on introduced fluorochromes, could enable cell-specific analyses and comparisons (Poulopoulos et al., 2019). Additionally, male and female animals were randomly used for HP-GC and DG-GC preparations without balanced sex representation, precluding conclusions about potential sex-dependent differences in hippocampal formation development.

In summary, our study provides a comprehensive characterization of the GC proteome originating from the whole HP and the DG during early postnatal development. Analysis of timepoint-specific and temporally regulated proteins revealed a clear shift in biological processes within GCs over time. For HP-GCs, P3 emerged as a particularly critical timepoint, with most proteins peaking in expression during this period. In contrast, DG-GCs exhibited a more prolonged developmental trajectory compared to HP-GCs, consistent with the known differences in maturation timelines between these regions. Our findings underscore the dynamic nature of the GC proteome during early postnatal hippocampal development and provide a foundation for understanding how local protein composition guides circuit assembly. The identification of temporally regulated disease-associated proteins highlights the potential importance of these developmental windows for understanding the origins of neurological disorders and suggests that this dataset may serve as a valuable resource for future studies investigating developmental mechanisms of disease pathogenesis.

## MATERIALS AND METHODS

### Animals

Mice belonging to the Odz3-tTa mouse line (originally published as MEC-13-53A in Blankvoort et al., 2018) were bred with a tetO-GCamP6 reporter mouse line (Blankvoort et al., 2018), both lines kindly donated by Clifford G. Kentros at Kavli Institute for Systems Neuroscience. Mice positive for both transgenes originating from this cross were used for experiments in Fig. 1. GC isolation and control samples were taken from wild-type mice (C57BL/6JBomTac, Taconic). All animals were housed in enriched environment cages in a reversed 12 h light/12 h dark cycle with humidity and temperature-controlled housing rooms. Food and water were provided *ad libitum*. Both male and female mice were used for experiments in this study; sex was not recorded or balanced across groups (see Discussion). All experiments were conducted in compliance with protocols approved by the Norwegian Food Safety Authorities and European Directive 2010/63/EU (FOTS ID 24847; 31016).

### Euthanasia

All animals were first anesthetized with isoflurane before being euthanized. Pups were euthanized by decapitation and adult animals with a lethal intraperitoneal injection of pentobarbital (100 mg/kg).

### Immunohistochemistry

After euthanasia, animals were transcardially perfused using 5-6 ml of phosphate-buffered saline (PBS) followed by 8-9 ml of 4% (w/v) paraformaldehyde (PFA) in PBS at a flow rate of 3.8-4.0 ml/min. Post-perfusion, brains were extracted and post-fixed in PFA for a minimum of 4 h, followed by 24 h in PBS. The brains were then sequentially incubated for 24 h each in 15% (w/v) and 30% (w/v) sucrose in PBS. Subsequently, the brains were embedded in optimal cutting temperature (OCT) mounting medium and rapidly frozen in a 70% (v/v) ethanol bath. Horizontal brain sections were prepared using a cryostat (CryoStar NX70, Thermo Fisher Scientific) at a thickness of 20 μm, directly mounted on adhesion slides (Superfrost Plus Adhesion Microscope Slides, Epredia) and stored at −20°C until further usage. For immunohistochemistry, the adhesion slides were thawed and framed using a PAP Pen (5 mm tip, Merck) before being rinsed in PBS for 10 min. Blocking was performed for 30 min in blocking solution [1× PBS, 0.1% (v/v) Triton X-100, 10% (v/v) donkey serum]. The slides were then incubated overnight at 4°C with the following primary antibodies: NeuN (guinea pig, anti-NeuN, Sigma Millipore, #ABN90P, 1:1000), GFP in GCamP6 (chicken, anti-GFP, Abcam, #AB13970, 1:1000; to detect GFP part of GCamP6) and Stx7 (rabbit, anti-Stx7, Synaptic Systems, #110072, 1:200). Antibodies were diluted in incubation buffer [1× PBS, 0.1% (v/v) Triton X-100, 1% (v/v) donkey serum]. The following day, slides were rinsed 6×5 min in PBS and incubated for 2 h with the following secondary antibodies: donkey anti-guinea pig 405 (Jackson ImmunoResearch, #706-475-148, 1:250), donkey anti-chicken 568 (Invitrogen, #A78950, 1:500) and donkey anti-rabbit 647 (Invitrogen, #A31573, 1:500), diluted in incubation buffer. After rinsing the slides 6×5 min in PBS, they were coverslipped with Flouromount-G (Invitrogen) and sealed with standard transparent nail polish.

### Confocal imaging and growth cones count

The HP and EC were imaged in 10× (Plan-Apochromat 10×/0.45 M27) and 40× magnification (Plan-Apochromat 40×/1.4 oil DIC M27) using the LSM 880 confocal microscope (Zeiss). Tiled *z*-stacks were acquired and a 3D image was reconstructed from the *z*-stack layers. In total, three horizontal sections from each of three animals (*n*=9) were chosen for imaging. Sections used for counting were a minimum of 150 μm apart. All images were processed in Zen software (blue edition, version 2.6.76, Carl Zeiss Microscopy, 2018) and further deconvoluted using Huygens Essential 23.10 software (Scientific Volume Imaging). Deconvoluted confocal imaging files containing the HP were uploaded to Neurolucida 360 (Micro Bright Field Bioscience) for analysis. To analyse the fluorescent intensity of invading EC reelin-expressing Layer 2 fibres and the amount of GCs in the HP, contours delineating the CA3 SLM, and the ML of the inner blade and of the outer blade of the DG, were created. GCs that were both positive for GFP and Stx7 were labelled with markers and counted. As non-consecutive slices were analysed, overcounting the *z*-axis was not relevant, and no correction was applied. For the quantification of the number of count markers and the fluorescent signal within the delineated areas, contours and counts were exported as xml files and quantified using a customized MatLab (R2023a version, Mathworks) script. Briefly, the Cartesian coordinates for contours and counts were extracted from the xml format Neurolucida 360 files and transformed into a Boolean mask fitted to match the size of the original reference picture on which the delineations were drawn. The masks were used to extract the fluorescence intensity values of the topologically corresponding voxels from the reference confocal image. A squared region with a standardized size (400×400 pixels) located in the weighted midpoint (centroid) of the DG was chosen as the background region. A threshold value was set as being the sum of the average value plus three times the standard deviation of fluorescence intensity of the background region for each image. The number of voxels within each contour (CA3 SLM, inner ML and outer ML) in which the fluorescence intensity exceeds the threshold value were counted (Fig. S1B). Voxel counts were then normalized to the total number of voxels for the corresponding delineated area. Outliers within the boxplots were identified based on the quartile method and labelled with asterisks. The script used for evaluation is available from https://github.com/Quattrocolo-Lab/krause-et-al-2026.

### Hippocampus and dentate gyrus microdissections

Animals at P1, P3 and P5 were euthanized and decapitated. The brains were promptly extracted from the skull, and the regions of interest were dissected on ice under a Stereo Microscope SZ16 (Olympic Life Sciences) in ice-cold growth cone homogenization (GCHO) buffer consisting of 4 mM Hepes, 0.32 M sucrose (pH 7.4) supplemented with 100 μl of 0.5 M EDTA stock and 100 μl Halt protease inhibitor cocktail per 10 ml (Thermo Fisher Scientific). First, the cerebellum was removed and the hemispheres separated by a midline incision along the longitudinal fissure. With the medial side facing up, the white matter was excised, exposing the HP. The HP was carefully flipped out of the cortex using a small paint brush or forceps. The fibres connecting the cortex to the HP were severed. For the DG samples, the HP was subsequently carefully flipped medial side up, and the DG was isolated by gently separating it from the CA, using as reference the visual gap between the DG and CA regions, along the septotemporal axis. The HP and DG samples were either frozen for RNA extraction the following day or immediately immersed in ice-cold GCHO buffer and stored on ice for GC preparation the same day.

### qRT-PCR

RNA was extracted from microdissected DG and HP P4 tissues using the NucleoSpin RNA/Protein kit (Machinery-Nagel) following the manufacturer's protocol. Reverse transcription of 100 ng of RNA into cDNA was performed using the Eurogentec RT-RTCK-05 Reverse Transcriptase Core kit 500. qRT-PCR was conducted in 20 μl reaction containing 10 μl SensIFAST SYBR mix, 2 μl forward and reverse primer mix (10 μM each), and 8 μl cDNA (1:10 diluted RT reaction), using StepOne Real-Time PCR system (Applied Biosystems). Cycler conditions included initial denaturation at 95°C for 2 min, followed by 40 cycles of denaturation at 94°C for 5 s, annealing at 60°C for 10 s, and extension at 70°C for 20 s. Cт values were averaged from technical duplicates, excluding values above 35. Relative gene expression was calculated using the 2(−ΔΔCт)-method (Schmittgen and Livak, 2008), normalized to *Gusb* (*Gus*; Xu et al., 2018). Primer specificity for housekeeping gene *Gusb* was confirmed through NCBI BLAST searches specific to *Mus musculus*. The dual specificity phosphatase 14 (*Dusp14*) was used as a marker for DG tissue, alongside three members of the Neurod family, a transcription factor family that is crucial in tissue development and maintenance (Tutukova et al., 2021): *Neurod1* and *Neurod4* showed specific expression for the DG, and *Neurod6* for the CA region. Further CA markers were *Meis2*, a homeodomain transcription factor, and *Tyro3*, which are adult CA markers (Hagihara et al., 2009). Primers, listed in Table 2, were designed using NCBI Primer-Blast with a target product length of 70-150 bp. Statistical significance of mRNA levels between CA and DG were detected by independent two-tailed Student's *t*-test.

**Table 2.** 5′→3′ sequences and function or tissue specificity of primers used for qRT-PCR

Function/specificity	Primer	5′→3′ sequence
Housekeeping gene	*mGus*-Fw	CCGACCTCTCGAACAACCG
	*mGus*-Rv	GCTTCCCGTTCATACCACACC
DG-specific genes	*mNeurod1*-Fw	TTCAAACACGAACCATCCGC
	*mNeurod1*-Rv	GTCTATGGGGATCTCGCAGC
	*mNeurod4*-Fw	CAGCCAAGGTACTCAGACCAG
	*mNeurod4*-Rv	GCAGTCAGTTCTACCCTGACC
	*mDusp14*-Fw	GCTTAGTCCTCCTCTCGGAA
	*mDusp14*-Rv	TTGGGAGCGGTGCTTGTTA
HP-specific genes	*mNeurod6*-Fw	GACGTCACTAGCAGATGGCA
	*mNeurod6*-Rv	TTTCAGGCTGAGTGTCGCAT
	*mMeis2*-Fw	CTGGCGAGATCACGATGACG
	*mMeis2*-Rv	AAGCTACGCTGTTGTCTAACC
	*mTyro3*-Fw	GCCTCCAAATTGCCCGTCA
	*mTyro3*-Rv	CCAGCACTGGTACATGAGATCA

Fw, forward; Rv, reverse.

### Growth cone preparation

The GCs were prepared as detailed in Lohse et al. (1996) and Pfenninger et al. (1983). Briefly, the microdissected tissue was homogenized in GCHO buffer using a glass-Teflon homogenizer coupled with Hei-TORQUE overhead stirrer (Heidolph) with 13 slow and even strokes at 900 rpm. The homogenate was transferred to a reaction tube and centrifuged for 15 min at 1700 ***g***, at 4°C (Eppendorf, Refrigerated Microcentrifuge 5426). The PNH was separated from the pellet and subsequently diluted with GCHO buffer to a final volume of 1.7 ml. A discontinuous sucrose gradient was layered in ultra-clear centrifugation tubes (5.6 ml, Beckman No: 344075) with three layers: a bottom cushion (4 mM Hepes, 2.5 M sucrose, pH 7.4), a middle layer (4 mM Hepes, 0.83 M sucrose, pH 7.4) and a top layer of the diluted PNH, all with a volume of 1.7 ml. The tube necks were sealed, and the gradient was centrifuged at 250,000 ***g*** for 50 min at 4°C using a Sorvall Discovery 100SE (Thermo Fisher Scientific) or Optima XE-90 (Beckman Coulter) equipped with a VTi50 Rotor (Beckman Coulter). Following centrifugation, GC particles (F1 fraction) were recovered from the interface between the middle and top sucrose layer using an 18-gauge needle and a 3 ml syringe. The F1 fraction was either transferred onto a polycarbonate membrane filter previously washed with PBS (Millipore, VCT02500, pore size 0.1 μm), filtered with Rotavac Valve Control vacuum pump (Heidolph, 591-00130-00-2), washed with PBS (4× with 1 ml of PBS) to remove potential proteomic contaminations, and stored at −80°C until analysis by LC-MS/MS or diluted 1:5 in PBS and pelleted at 12,000 ***g*** for 5 min, and resuspended in in PBS to remove potential proteomic contaminations, before being mixed with 4× Laemmli buffer β-mercaptoethanol and incubated at 95°C for 5 min for subsequent western blot analysis.

### Western blot

GC preparation was validated by western blot. F1 fractions were prepared for western blot analysis as described above (‘Growth cone preparation’). Synaptosome and total hippocampal protein extract control samples were prepared following the synaptosome preparation by Dunkley et al. (2008) and using RIPA buffer (Thermo Fisher Scientific) extraction according to the manufacturer's protocol. Both types of control samples were denatured with Laemmli buffer as described above. Protein concentrations were detected using the G-Biosciences Compatible Lowry assay, in which 1 μg of each sample was separated via SDS-PAGE on 8-16% Mini-PROTEAN TGX Stain-Free gels (Bio-Rad) under the manufacturer's 200 V standard protocol. Proteins were transferred to PVDF membranes, blocked with TBS-T [1× TBS, 0.1 (v/v) % Tween-20] containing 5% (w/v) skim milk powder for 1 h at room temperature, and probed overnight at 4°C with primary antibodies diluted in TBS-T with 1% (w/v) skim milk powder. The primary antibodies used were: rabbit anti-growth associated protein-43 (1:1000, Merck, #AB5220), rabbit anti-synaptophysin antibody (1:2500, Abcam, #14692), rabbit anti-Map2 polyclonal antibody (1:1000, Proteintech, #17490-1-AP), mouse anti-PSD95 antibody [K28/43] synaptic marker (1:2500, Abcam, #192757), and mouse anti-β-actin loading control monoclonal antibody (BA3R) (1:1000, Invitrogen, #MA5-15739). After washing (3×10 min in TBS-T), the membrane was incubated for 1 h 30 min at room temperature with the following HRP-conjugated secondary antibodies: goat anti-rabbit IgG (H+L) (1:2500, Promega, #W4011) and rabbit anti-mouse IgG (H+L) (1:5000, Invitrogen, #31450), diluted in TBS-T with 1% skim milk powder. The membrane was washed (3×10 min in TBS-T), incubated in ECL solutions (Clarity Western Peroxide Reagent and Enhancer Reagent, Bio-Rad) for 2 min, and chemiluminescence was detected using the ChemiDoc XRS+ system and ImageLab software (Bio-Rad).

### LC-MS/MS

Mass spectrometry sample analysis was performed by the Proteomics and Modomics Experimental Core Facility (PROMEC, NTNU). Tryptic samples were prepared from polycarbonate filters by first recovering the GCs in 600 μl 1% SDC, 100 mM Tris-HCl (pH 8.5), 10 mM TCEP, 40 mM CAA and sonicating for 30 min at 70°C. Afterwards, 600 μl 0.1 M ammonium bicarbonate, 0.5 μg trypsin were added and the samples digested overnight at 37°C. Peptides were desalted using C18 spin columns, dried in a speedvac centrifuge (at 240 ***g***) and resuspended in 0.1% formic acid before MS analysis. LC-MS/MS were performed on a timsTOF Pro (Bruker Daltonics) connected to a nanoElute (Bruker Daltonics) HPLC. The peptide solution was injected, and separation was carried out using a Pepsep 25 (150 μm×25 cm) column with running buffers A (0.1% formic acid) and B (0.1% formic acid in acetonitrile) with a gradient from 0% B to 40% B for 75 min. The timsTOF instrument was operated in DDA PASEF mode with 10 PASEF scans per acquisition cycle and accumulation and ramp times of 100 ms each. The ‘target value’ was set to 20,000 and a dynamic exclusion was activated and set to 0.4 min. The quadrupole isolation width was set to 2 Th for m/z<700 and 3 Th for m/z>800.

### Proteomic data analysis

TimsTOF DDA raw data files were interpreted using MaxQuant software v. 2.6.1.0 (Cox and Mann, 2008). Proteins were identified with one unique peptide and an FDR of 0.01 using the mouse proteome including isoforms from Uniprot (downloaded: 14.03.2024) as a reference dataset. Analysis parameters were Trypsin/P as a digestion enzyme; a maximum of two missed cleavages were allowed; six amino acids were used as minimum peptide length; oxidation (M), acetyl (protein N-term), and deamidation (NQ) were set as variable modifications. LFQ was performed with match between runs enabled using MaxQuant standard settings. PTXQC R package (Bielow et al., 2016) was used to quality control results generated by MaxQuant. All further downstream analyses were conducted using a custom R script (R v. 4.2.3, script available from https://github.com/Quattrocolo-Lab/krause-et-al-2026). All proteins labelled as ‘potential contaminants’, ‘only identified by site’, and ‘reverse’ were removed from the dataset. Proteins were considered as identified for a timepoint and tissue type if three out of five biological replicates showed an LFQ intensity value. GO term analyses were performed using ClusterProfiler v. 4.4.4 (Xu et al., 2024). GO term results were manually curated and redundant terms triggered by identical proteins removed. KEGG pathway analyses for mouse protein terms were performed using the online platform ShinyGO 0.85 (Ge et al., 2020). For single and multi-series time course analyses the package maSigPro v. 1.68.0 (https://bioconductor.org/packages/maSigPro) was used with a quadratic regression model (degree=2) and the parameters Q=0.05, Benjamini–Hochberg correction for multiple testing adjustment, and a minimum of four observations for single series time course analysis using P1 as reference and the parameters Q=0.05, Benjamini–Hochberg correction for multiple testing adjustment, and a minimum of six observations for the multi-series time course analysis with the hippocampal data as reference dataset. Human orthologous genes were identified using orthogene v. 1.2.1 (http://bioconductor.org/packages/orthogene/). Disease terms were identified by using DOSE v. 3.22.1 (Yu et al., 2015). All plots were generated in R using the packages ggplot2 v. 4.0.0 (https://cran.r-project.org/web/packages/ggplot2/citation.html), ggVennDiagram v. 1.5.2 (https://github.com/gaospecial/ggVennDiagram), ggvenn v. 0.1.10 (https://cran.r-project.org/web/packages/ggvenn/index.html), ggalluvial v. 0.12.5 (https://github.com/mbojan/alluvial) and ComplexHeatmap v. 2.14.0 (Gu et al., 2016). Raw data are available at the ProteomeXchange consortium (http://proteomecentral.proteomexchange.org) submitted via the PRIDE partner repository as PX Partial with the dataset identifier PXD076616 (Deutsch et al., 2017; Perez-Riverol et al., 2022).

## Supplementary Material



10.1242/develop.205342_sup1 Supplementary information
