# Supplementary material for: Proteomic profile of hippocampal growth cones through early postnatal development
Source: Development. 2026 Jun 22;153(12):dev205342. doi: 10.1242/dev.205342 (PMC13354955; doi:10.1242/dev.205342)
Supplement: Supplementary information [file develop-153-205342-s1.pdf]

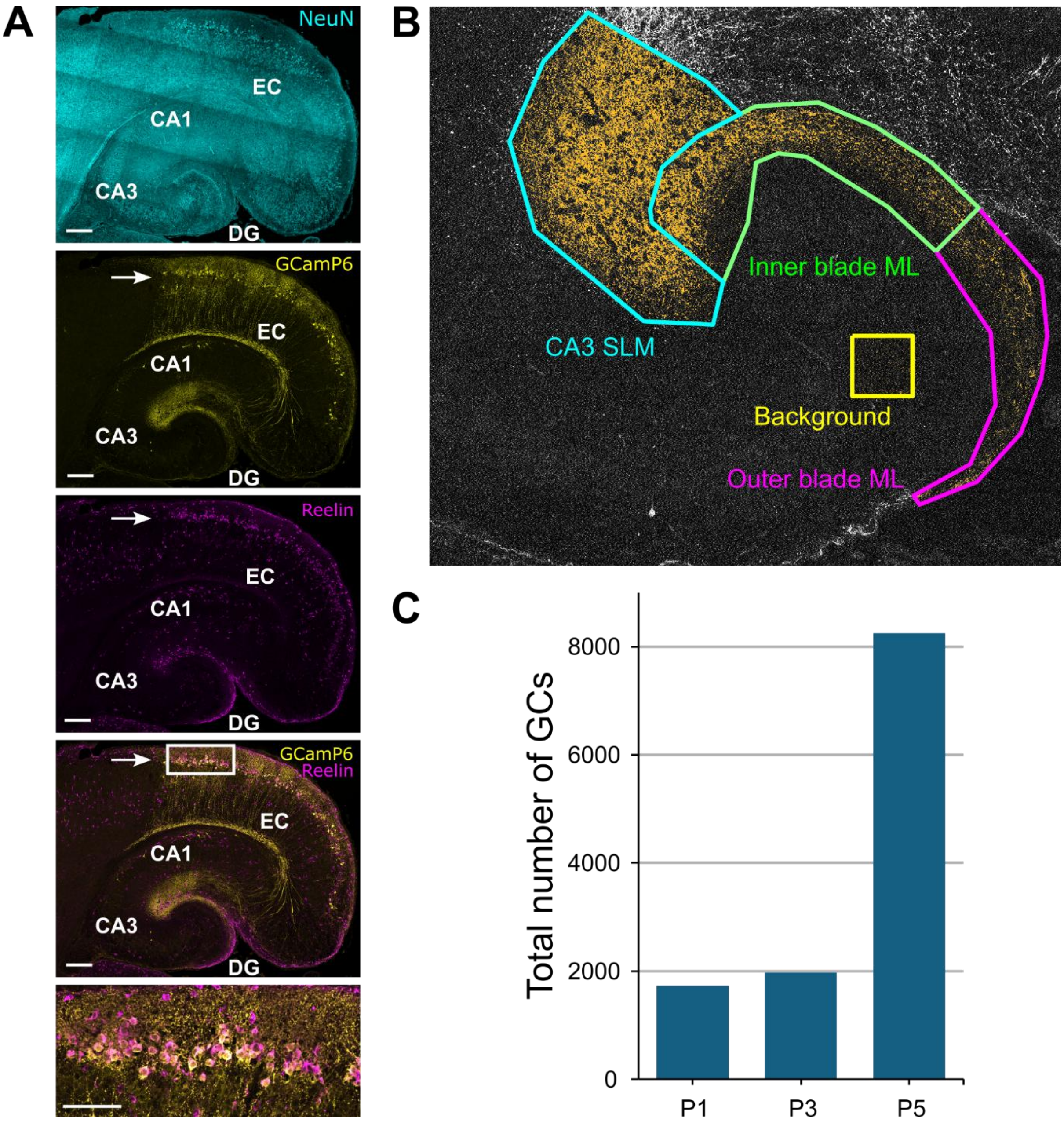

Fig. S1. A) Confocal image of horizontal sections Odz3-tTa;TetO-GCamP6 animal at P5. Overview of the entorhinal-hippocampal region in the top image visualized with NeuN staining in turquoise. GCamP6 staining in yellow (second image) labelled Layer 2 cells of the EC (indicated by arrow). All Reelin-positive cells are shown in the third image in violet. The fourth image represents a merged image of the GCamP6 and Reelin staining clearly showing that the entorhinal Layer 2 cells are Reelin-positive. The last image shows a zoom in on entorhinal Layer 2 cells. Scalebar: 200 µm and 100 µm in zoom in. B) Representative depiction on the thresholding approach for the fluorescent signal evaluation for Fig. 1B. Shown is one layer of a z-stack P5 image with the delineated areas (CA3 SLM, Inner blade ML, Outer blade ML, and Background) in greyscale. All pixels exceeding the threshold and contribute to the analysis within the contours are depicted in yellow. C) Total number of GCs counted in nine sections at P1, P3, and P5.

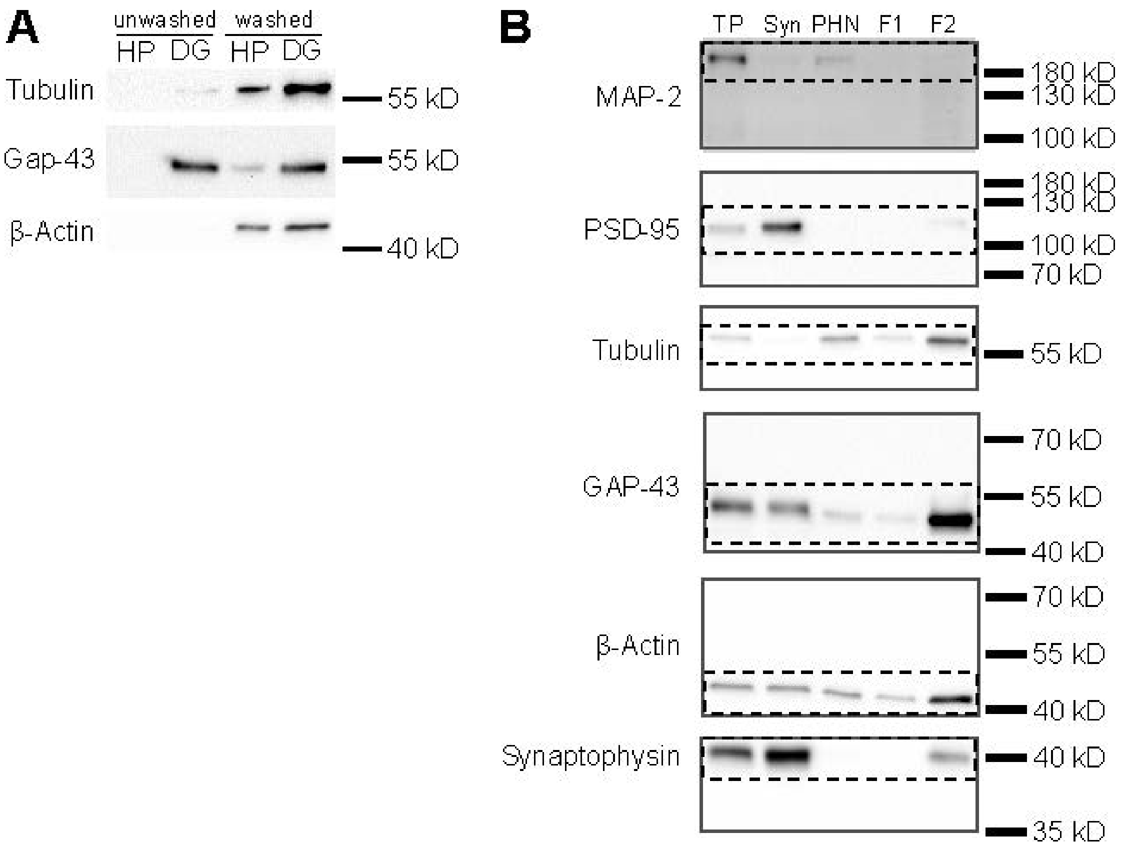

Fig. S2. A) Western Blot control of unwashed and washed GC F1 fraction from HP and DG at P5. Loaded were 1 µg of protein per lane. GC associated proteins Tubulin, Gap-43, and β-Actin can be clearly detected in the washed preparations and only to some extent or not at all in the unwashed preparations confirming that the washed F1 fractions used in our study have an increased amount of GCs compared to an unwashed preparation. B) Western Blot validation of total HP-GC preparation at P4. Grey boxes indicate the original size of the detected membrane, and the black dashed boxes are the part of the blot depicted in Fig. 2C.

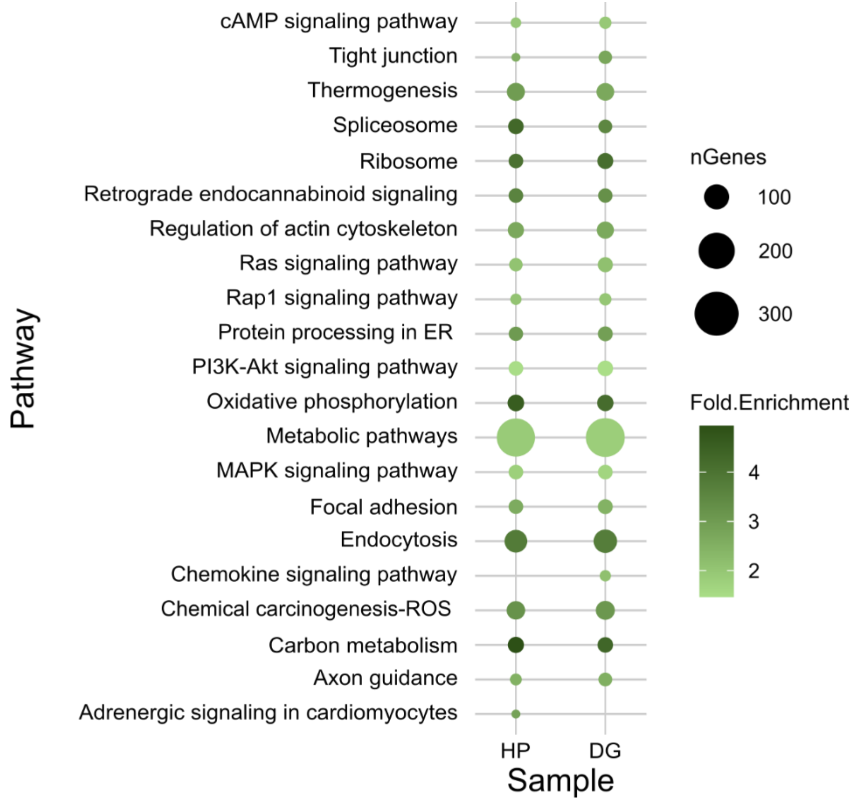

Fig. S3. Comparison of Top20 KEGG pathway results for the shared proteome of HP-GCs and DG-GCs.

Development • Supplementary information

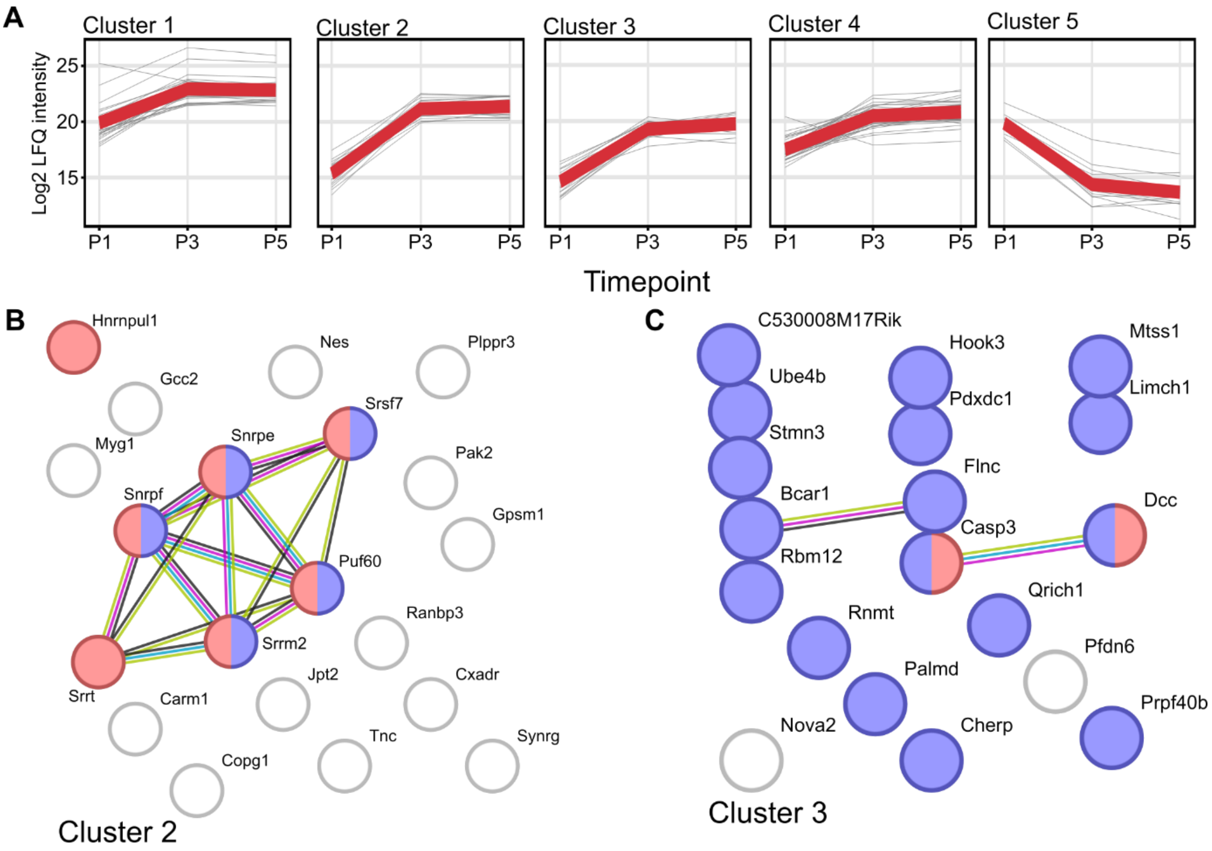

Fig. S4. A) Averaged (red curves) and protein individual (grey curves) changes in Log2 LFQ intensity for each single time series clusters based on Fig. 4D. All proteins identified as temporally significantly changing have an increasing (Cluster 1 to 4) or decreasing (Cluster 5) trend. STRING network for Cluster 2 (B) and 3 (C) of single-time series analysis clusters for the DG-GC. B) Red highlights the proteins that belong to the functional cluster for *RNA processing* including *Splicing via spliceosome* (labelled in blue). C) Only two interacting protein pairs were identified in this cluster. Interestingly almost all proteins included in this cluster are phosphoproteins (highlighted in blue), except NOVA2 and PFDN6. Red highlighted network represents the pathway *Caspase activation via Dependence Receptors in the absence of ligand* and contains the GC marker DCC.

Development • Supplementary information

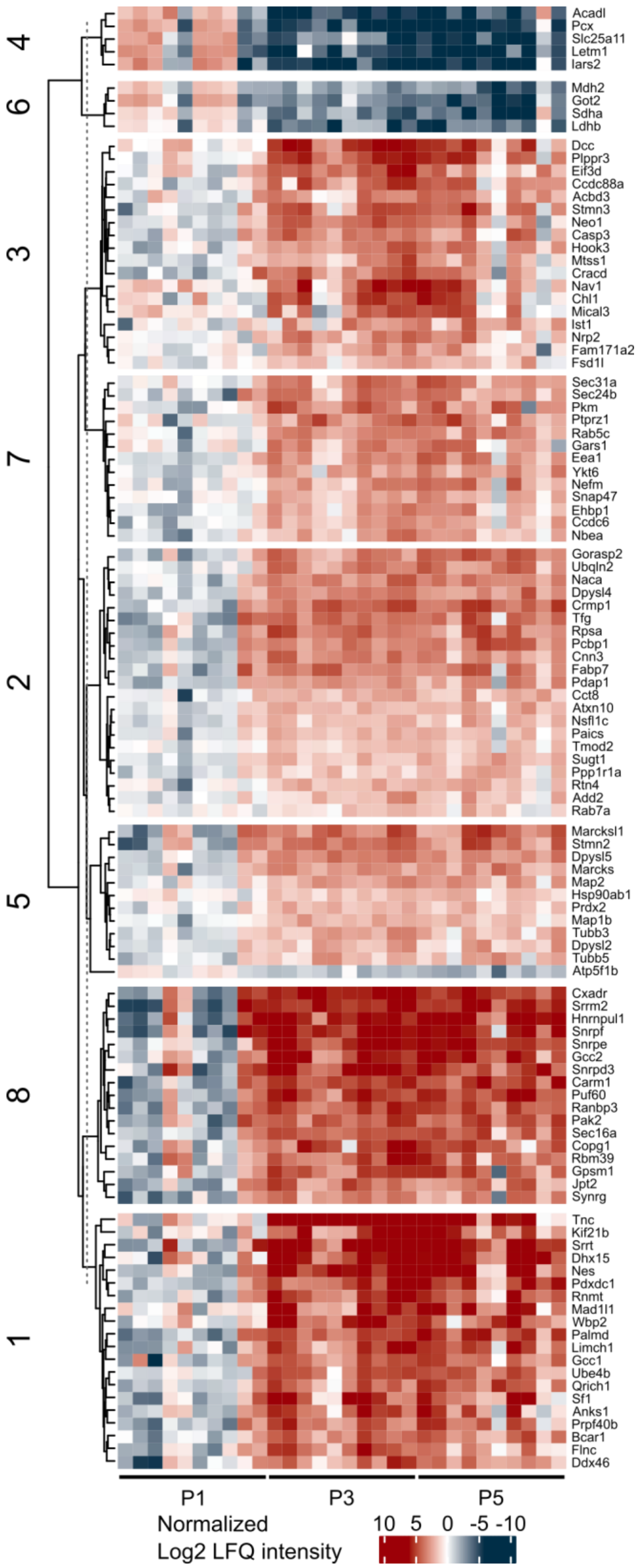

Fig. S5. Heatmap of significant proteins identified in the multi-time series analysis with HP as control. Depicted are the normalized individual Log2 LFQ intensities and their cluster association. Protein names and their corresponding clusters can also be found in Table S1.

Development • Supplementary information

Table S1. List of proteins in alphabetic order identified in time series analysis in Figs 3E, S4D, and 6A with their respective cluster.

**Single-time series analysis**				**Multi-time series analysis**	
**HP**		**DG**		**DG vs HP**	
Gene name	Cluster	Gene name	Cluster	Gene name	Cluster
Bzw2	2	Acadl	5	Acadl	4
Cspg4	1	Acadm	5	Acbd3	3
Dcc	1	Add2	4	Add2	2
Dhx15	2	Anxa5	4	Anks1	1
Hnrnph2	2	Arl3	4	Atp5f1b	5
Nav1	1	Atp5f1b	1	Atxn10	2
Nudcd3	2	Atxn10	1	Bcar1	1
Ppp2r2a	1	Bcar1	3	Carm1	8
Rab5a	4	Carm1	2	Casp3	3
Rab5c	4	Casp3	3	Ccdc6	7
Sec23a	4	Ccdc6	4	Ccdc88a	3
Sec31a	4	Cdkn1b	4	Cct8	2
Snrpe	3	Cherp	3	Chl1	3
Tnc	1	Ciapin1	4	Cnn3	2
Wbp2	2	Clip1	4	Copg1	8
		Cnn3	1	Cracd	3
		Copg1	2	Crmp1	2
		Copz1	4	Cxadr	8
		Cracd	3	Dcc	3
		Crmp1	1	Ddx46	1
		Crmp1	1	Dhx15	1
		Cs	5	Dpysl2	5
		Cxadr	2	Dpysl4	2
		Dcc	3	Dpysl5	5
		Dpysl4	1	Eea1	7
		Dpysl5	1	Ehbp1	7
		Dync1i2	4	Eif3d	3
		Dynlrb1	1	Fabp7	2
		Eea1	4	Fam171a2	3
		Ehbp1	4	Flnc	1
		Etfb	5	Fsd1l	3
		Fabp7	1	Gars1	7
		Flnc	3	Gcc1	1
		Fsd1l	4	Gcc2	8
		Gcc2	2	Gorasp2	2
		Gng2	1	Got2	6
		Gorasp2	4	Gpsm1	8
		Gpsm1	2	Hnrnpul1	8
		Hnrnpul1	2	Hook3	3

Development • Supplementary information

Single-time series analysis				Multi-time series analysis	
HP		DG		DG vs HP	
		Hook3	3	Hsp90ab1	5
		Hspa4l	5	Iars2	4
		Iars2	5	Ist1	3
		Jpt2	2	Jpt2	8
		Limch1	3	Kif21b	1
		Map1b	1	Ldhb	6
		Map2	1	Letm1	4
		Marcks	1	Limch1	1
		Mtss1	3	Mad1l1	1
		Myg1	2	Map1b	5
		Naca	4	Map2	5
		Nbea	4	Marcks	5
		Nefm	4	Marcksl1	5
		Neo1	4	Mdh2	6
		Nes	2	Mical3	3
		Nova2	3	Mtss1	3
		Nrp2	4	Naca	2
		Nsfl1c	1	Nav1	3
		Nudc	1	Nbea	7
		Nudt5	4	Nefm	7
		Paics	4	Neo1	3
		Paip1	4	Nes	1
		Pak2	2	Nrp2	3
		Palmd	3	Nsfl1c	2
		Pcbp1	1	Paics	2
		Pcx	5	Pak2	8
		Pdap1	4	Palmd	1
		Pdcd5	1	Pcbp1	2
		Pdhx	5	Pcx	4
		Pdxdc1	3	Pdap1	2
		Pfdn6	3	Pdxdc1	1
		Pkm	4	Pkm	7
		Plppr3	2	Plppr3	3
		Plxna2	4	Ppp1r1a	2
		Ppp1r1a	1	Prdx2	5
		Prdx2	1	Prpf40b	1
		Prpf40b	3	Ptprz1	7
		Ptprz1	4	Puf60	8
		Puf60	2	Qrich1	1
		Qrich1	3	Rab5c	7
		Rab5c	4	Rab7a	2
		Ranbp3	2	Ranbp3	8

Development • Supplementary information

Single-time series analysis				Multi-time series analysis	
HP		DG		DG vs HP	
		Rbm12	3	Rbm39	8
		Rnmt	3	Rnmt	1
		Rpsa	1	Rpsa	2
		Sdha	5	Rtn4	2
		Sec16a	4	Sdha	6
		Sec24b	4	Sec16a	8
		Slc25a11	5	Sec24b	7
		Snap29	4	Sec31a	7
		Snap47	4	Sf1	1
		Snrpe	2	Slc25a11	4
		Snrpf	2	Snap47	7
		Snx3	1	Snrpd3	8
		Srrm2	2	Snrpe	8
		Srrt	2	Snrpf	8
		Srsf7	2	Srrm2	8
		Stmn1	1	Srrt	1
		Stmn3	3	Stmn2	5
		Sugt1	1	Stmn3	3
		Synrg	2	Sugt1	2
		Tfg	1	Synrg	8
		Timm44	5	Tfg	2
		Tjp1	4	Tmod2	2
		Tmod2	1	Tnc	1
		Tnc	2	Tubb3	5
		Tnik	4	Tubb5	5
		Tubb3	1	Ube4b	1
		Ube2m	4	Ubqln2	2
		Ube4b	3	Wbp2	1
		Ubqln2	1	Ykt6	7
		Ykt6	4		

Table S2. Human orthologues genes represented in our dataset that are associated with neurological and neurodegenerative diseases. The subset of time-regulated disease associated orthologous genes are written in bold. Table depends on disease term enrichment analysis and selection of relevant diseases.

Disease	Human orthologue genes of proteins in dataset
Alexander disease	GFAP, MTOR
Alzheimer's disease	ANK3, ACHE, LTF, UBXN4, QPCT, SERPINA3, APBA2, BAX, APP, CYP2D6, TFCP2, DLG4, KLC1, AQP4, NPTXR, PLTP, AGRN, HSPG2, APBA1, HTRA2, CSF1, GSK3A, ADAM17, ADAP1, ITSN1, GSK3B, COL18A1, BAG1, NOS1, CTSD, DNM2, SP1, CP, PAK1, BSG, BIN1, FAAH, FLOT1, SEMA4D, GSTO1, CAPN1, FOLH1, ADAM10, SNCA, HMOX2, SORL1, HNRNPA2B1, COMT, STX8, ITM2B, C3, GFAP, PRNP, TTR, HSP90AA1, APOE, SOD1, NEFL, ITGB1, ACE, SOD2, LPL, FABP3, CDK1, CAMK2A, GSN, GLUL, CHGB, ENO2, GSTP1, F2, HSPA5, CST3, PTGS1, CRYAB, CHGA, APEX1, GRN, AKT1, MAP2K1, MIF, APOC1, PRDX1, FYN, PURA, SLC1A2, ITGAV, **CDKN1B**, ALDH2, CDK5, PSEN1, CAV1, S100B, ARSA, CAST, **RAB7A**, PON1, **STMN1**, PTPN5, ARPP19, NCSTN, NPY, FGF1, VSNL1, GNAS, PPP3CA, RACK1, NOS3, **NACA**, **CASP3**, APOA1, VCP, AQP1, CTNNB1, ATP5F1A, CKB, APLP2, CLU, IGF2R, GRM2, WWC1, SLC30A1, RELN, SLC6A4, MEF2A, DYRK1A, MME, MAPK3, MAP2K2, MAOA, SQSTM1, PRDX6, TTBK1, EIF2S1, DAPK1, SLC18A2, RPS6KB1, MAOB, PTGES2, BACE1, C1R, IDE, FGB, BLMH, GGA1, HNMT, CBS, TARDBP, TF, PARP1, MTHFD1, HSD17B10, **RTN4**, **RAB5A**, PPID, CSNK1D, RCAN1, MTOR, DKK3, PIN1, DBN1, PCSK1N, GPC1, UCHL1, STUB1, CYP46A1, DDX39B
Amyotrophic lateral sclerosis (ALS)	ELP3, SPAST, APP, CCS, KIF1B, SETX, KIF3A, AIFM1, IGFBP2, HTRA2, TMEM106B, HDAC11, GSK3A, SMAD2, DCTN1, UBE2H, GSK3B, GRM5, NOS1, CP, SLC16A2, C3, APOE, SOD1, NEFL, P4HB, CTSB, CHGB, GSTP1, NEFH, CST3, CHGA, APEX1, GRN, MMP2, STAT3, SLC1A2, IGFBP3, PON1, FUS, IDI1, PTPA, GABRA1, KIFAP3, PKN1, **CASP3**, VCP, SCG2, VGF, GRM2, UNC13A, PON2, PON3, NIPA1, CHMP2B, IFT74, TAF15, OPTN, FLAD1, **CSPG4**, ALS2, TARDBP, PARP1, KARS1, **RTN4**, **RAB5A**, TXNRD1, PIN1, VAPA
Ataxia telangiectasia	BAX, RPS6KA3, MCM3, H2AX, AKT1, STAT3, PPP1CC, **CASP3**, MCM2, MCM7, UPF1, MTOR
Autism spectrum disorder	MEF2C, EGF, GRIK2, DOCK4, AQP4, SCN2A, NLGN3, SCN1A, MACROD2, SCN3A, GABRA3, APC, AGAP1, UBE2H, CADPS2, PCDH19, NLGN1, CNTNAP2, GABRB3, CNTN4, CTSD, GRIP1, UBE3A, ARX, **NRP2**, ITGB3, DDC, COMT, ADA, APOE, GPX1, CALB1, NTRK2, GJA1, GABRA2, AKT1, MIF, GRIN2A, GRM8, GAD1, GABRB1, PON1, ADSL, CD38, ABAT, GABRA1, RAB3A, PRKCB, **CASP3**, CDH8, CTNNB1, TSPAN7, SHANK3, SEZ6L2, RELN, SLC6A4, HEPACAM, MAOA, GABBR2, WNK3, SLC25A12, GABRA5, FAM120C, TPH2, CADM1, ATP1A1, MARK1, NDUFA5, GLO1, NRXN1, TSC1, **NBEA**, SCAMP5, COPG2, GABBR1, STK39
Canavan disease	ASPA
Charcot-Marie-Tooth disease	MED25, DNM2, GDAP1, NEFL, HSPB1, LMNA, NDRG1, MFN2, LRSAM1, AARS1, KARS1, **GARS1**, MTMR2
Cockayne syndrome	LMNA
Early myoclonic encephalopathy	SCN8A, SCN2A, SCN1A, NDUFA1, BRD2
Encephalitis	LTF, LGI1, APP, **TJP1**, RIMS2, MAP3K5, CNTNAP2, APOE, ENO2, PPIB, GRN, VCAM1, SSB, PTX3, CDK5, FUBP1, CLDN11, DDX42, TLR3
Epilepsy syndrome	MEF2C, CDKL5, LGI1, SCN8A, TLN2, ANXA7, RALBP1, NEDD4L, SCN2A, GPHN, GOSR2, SCN1A, KCNQ2, PCDH19, ATP6V0C, LAMB1, CNTNAP2, GABRB3, NOS1, CTSD, SCN3B, CACNB4, **DPY**SL2, ARX, SCARB2, ATN1, NDUFA1, SERPINI1, HCN2, HCN1, C3, GFAP, PRNP, APOE, SOD1, KCNA1, SLC2A1, GSTP1, CST3, SSTR2, PVALB, GRIN2A, SLC1A2, SYT1, GAD1, GAD2, SLC1A3, ARF6, GABRA1, GABRB2, **CASP3**, NUCB2, SCN1B, EGFR, AQP1, UBA1, CALB2, TBC1D24, ERMN, RELN, SLC6A4, CSTB, ATP1A2, EIF2S1, BRD2, SEZ6, GABBR2, DAPK1, RCN2, LGI2, AP3M2, HIP1, PNPO, WASL, L2HGDH, **RTN4**, ARPC2, EPHX1, ALDH7A1, SV2A, KCNJ10, RHOA, PRODH, GABBR1
Hereditary spastic paraplegia	KIF5A, HSPD1
Huntington's disease	ARFGEF2, CP, FAAH, ATN1, SOD1, AKT1, MMP2, GRIN2A, CNR1, CDK5, NPY, CKB, KPNA1, MAOB, TCERG1, DNAJB2, UCHL1
Infantile epileptic encephalopathy	SCN1A
Leigh disease	ATP5PF, NDUFAF2, SRSF2, NDUFS1, NDUFS7
Lesch-Nyhan syndrome	HPRT1, APRT
Lewy body dementia	APP, CYP2D6, AQP4, TRIM9, SNCA, APOE, GPX1, CST3, GRN, RPH3A, PSEN1, NOS3, AQP1, MME, GBA2, SLC18A2, MAP1LC3A, SNCB, TARDBP, SNCG
Neuronal ceroid lipofuscinosis	SOD2

Development • Supplementary information

Niemann-Pick disease	APOE, NPC2
Parkinson's disease	ACHE, SCN8A, CYP2D6, BCL2L1, LINGO1, HTRA2, MAP3K5, SPR, GIGYF2, CP, CAPN1, NDUFA1, SNCA, COMT, C3, APOE, SOD1, ACE, GPX1, CALB1, NTRK2, PRDX3, TGM2, GRN, PVALB, EPHX2, HSPA9, CNR1, GAD1, GAD2, S100B, PON1, PSMC4, SST, YWHAZ, **CASP3**, PLA2G6, PSMB4, HS1BP3, FUBP1, LRRK1, SLC6A4, XIAP, FADD, **PRDX2**, MEF2D, SQSTM1, NQO1, SLC18A2, MAOB, IDE, SNCB, TARDBP, TF, PARP1, PARK7, EPHX1, NDUFV2, SYT11, UCHL1
Pick's disease	APP, SNCA, APOE, PRDX3, PRDX1, PSEN1, **PRDX2**, MAOA, SQSTM1, PRDX6, PIN1, PCSK1NAlsl
Prion disease	ACHE, APP, AQP4, CTSD, ADAM10, PRNP, CHGB, NFKB1, CHGA, HSPD1, AQP1, **NES**, TARDBP
Rett syndrome	CDKL5, FXYD1, APOE, PRPF40A
Sandhoff disease	SNCB
Spinal muscular atrophy	SYNCRIP, PLS3, NOLC1, HSPG2, TRA2B, SNCA, UBA1, KDSR, SCO2, **GARS1**
Spinocerebellar ataxia	CNTN4, ATN1, ITPR1, **ATXN10**, PRKCG, SPTBN2, ASIC1, AFG3L2, TARDBP, STUB1
Unverricht-Lundborg syndrome	SCARB2
Wolfram syndrome	COMT, WFS1
X-linked disease	PTK2, F5, RPS6KA3, F2, ACTR2, GABRA1, WAS, OCRL, WASF1, ACTR3, NCK1
Zellweger syndrome	PEX5

Development • Supplementary information
