# Supplementary material for: Proteomic profile of hippocampal growth cones through early postnatal development
Source: Development. 2026 Jun 22;153(12):dev205342. doi: 10.1242/dev.205342 (PMC13354955; doi:10.1242/dev.205342)
Supplement: Supplementary file 1 [file develop-153-205342_review_history.pdf]

Proteomic profile of hippocampal growth cones through early postnatal development

Maike Krause, Kamilla Aase Kronberg, Paulo J. B. Girão, Lara Sophie Strohmeier and Giulia Quattrocolo

DOI: 10.1242/dev.205342

Editor: Debra Silver

Review timeline

Original submission:	3 November 2025
Editorial decision:	15 December 2025
First revision received:	8 April 2026
Editorial decision:	5 May 2026
Second revision received:	21 May 2026
Accepted:	25 May 2026

Original submission

First decision letter

MS ID#: dev.205342

MS TITLE: Proteomic profile of hippocampal growth cones through early postnatal development

AUTHORS: Maike Krause; Kamilla Aase Kronberg; Paulo J. B. Girão; Lara Sophie Strohmeier; Giulia Quattrocolo

Dear Dr Quattrocolo,

I have now received all the referees' reports on the above manuscript, and have reached a decision. The referees' comments are appended below.

As you will see, the referees express considerable interest in your work, but have some significant criticisms and recommend a substantial revision of your manuscript before we can consider publication. Of note the reviewers indicate the need for additional analyses, as well as key modifications to the presentation and text. If you are able to revise the manuscript along the lines suggested, I will be happy to receive a revised version of the manuscript. Your revised paper will be re-reviewed by one or more of the original referees, and acceptance of your manuscript will depend on your addressing satisfactorily the reviewers' major concerns. Please also note that Development will normally permit only one round of major revision. If it would be helpful, you are welcome to contact us to discuss your revision in greater detail. Please send us a point-by-point response indicating your plans for addressing the referees' comments, and we will look over this and provide further guidance.

Please attend to all of the reviewers' comments and ensure that you upload both a 'clean' version of your Word file, along with a highlighted version clearly showing where you have made changes in the revised manuscript. Please avoid using 'Tracked changes' in Word files as these are lost in PDF conversion. I should be grateful if you would also provide a point-by-point response detailing how you have dealt with the points raised by the reviewers in the 'Response to Reviewers' box. If you do not agree with any of their criticisms or suggestions please explain clearly why this is so.

© 2026. Published by The Company of Biologists under the terms of the Creative Commons Attribution License (https://creativecommons.org/licenses/by/4.0/).

Reviewer 1

SUMMARY OF THE ADVANCE MADE IN THIS PAPER AND ITS POTENTIAL SIGNIFICANCE TO THE FIELD

Krause et al. address an important question about local proteome dynamics in the growing axon during brain development. The authors isolate growth cones of entorhinal cortex neurons at three timepoints and analyze protein homogenates by mass spectrometry.

SUGGESTIONS TO AUTHORS

What is well done:

- I am convinced that proteomics is central for understanding neurodevelopment, especially given the dominance of transcriptomics. The authors should make a stronger point that their methodology stands out, and anchor it with seminal proteome work from the Macklis, Silver, Miller, Kraushar, Barna and Tarabykin labs.

What needs revising:

- The abstract needs a full rewrite. It repeats results rather than offering a conceptual, thoughtful synthesis of the findings. Sentences such as "This earliest timepoint seems to be characterized by a high metabolic demand" should be removed, as they add no real information. A short explanation of EC layer 2 Reelin axons would also help readability.

- Abbreviation use is inconsistent. Sometimes terms are defined and sometimes not. Please revise.

- The sentence "Notably, we identified multiple proteins containing nuclear localization sequences…" should be removed. It is speculative and unsupported. The presence of an NLS in a sequence does not establish function.

- The references throughout need revision. For example, the mention of local protein expression (line 64-65) cites only a small fraction of relevant work. Please include newer evidence showing that local protein synthesis and degradation are key molecular drivers in development. The rationale of studying hippocampal growth cones is also not explained (line 68-69).

- Figure 1: zoom in on the quantified structures shown in the bar graphs in panel B; include these as insets in panel A. Panel C needs arrowheads. The legend must specify what is plotted, what the bars represent (mean?), and what the error bars denote. Report the statistical tests used.

- The mouse model is not described. Please explain the rationale for the transgenic line and what this line is. Other than visualizing GCs, it does not really serve the purpose - the reader is inclined to think some sort of fluorescence-based isolation would follow.

- Line 114 is unclear. The chosen timepoints appear arbitrary. Please explain the rationale, including the "critical window" referenced.

- Line 115 - remove "elegant."

- What is the contribution of Odz3-expressing Reelin cells to EC neuronal cell types, and why are these cells important?

- Line 121 - why is the GCaMP6 line used to label axons? Could lower fluorescence at P1 reflect reduced Ca^{2+} waves rather than lack of invasion?

- Line 129 - avoid wording like "well-known."

© 2026. Published by The Company of Biologists under the terms of the Creative Commons Attribution License (https://creativecommons.org/licenses/by/4.0/).

- Line 131 - the listed numbers of GCs counted are confusing. Is this per animal, per hippocampus? What does this number represent?

- Given that EC laminae project to different hippocampal regions (ML, SML), could the target region itself explain local proteome differences? If synaptic targets differ, presynaptic composition would be expected to differ as well. What is the influence of neuronal fate on growth cone composition?

- Figure 2: the microdissection validation is unclear. This step is critical to confirm that the proteomics reflect region-specific material. qRT-PCR alone does not validate this (especially with the observed data spread). Why not perform histology or ISH on dissected regions? In panel C, why is no presynaptic marker included, given that it should be enriched in GC fractions? Also, F1 is used for proteomics, yet the WB shows enrichment of GAP-43 in F2. Please clarify.

- The biochemical preparation of growth cones is confusing. Can the authors visualize GCs by microscopy and exclude other subcellular contaminants? If not, the fraction should be named differently.

- Line 172 - provide the exact ages of animals used for each fraction. Are all fractions in Fig. 2C age-matched? If not, the comparison is not meaningful.

- Line 184 - the sample description reads as if one animal generated five technical replicates. This design is not acceptable; replicates should be biological, from age-matched animals. How does sex contribute to sample preparation? How many male and female animals were used?

- Figure 3: did the authors validate the proteomic findings? For example, if P1 GCs are enriched for mitochondria (line 208) as compared to other stages, you could image mitochondria at P1, P3 and P5, similar as done on Fig. 1.

- Line 215 - references again need revision. For instance, Harnett et al., 2022 should be cited for tRNA in development.

- Figure 3E: why are only some proteins shown on the heatmap instead of full cluster contents? GO terms should be run for each cluster.

- The Fig. 3 data are problematic. The GC proteomes are not normalized to global protein levels in the region or to nuclear fractions. This likely explains the apparent spike in protein abundance at P3 across clusters. This should be considered in the interpretation. Including EC proteomes at P1, P3 and P5 might help.

- The results section needs reconceptualization. Pages 9-13 read as a loose walk-through of GO terms rather than a coherent narrative. What was the aim of the analysis? What was the original aim of the authors? Some experimental validation of highlighted processes (e.g., line 239-240) would help and I strongly encourage authors to follow-up.

- Part 6 lacks focus and feels out of place. It discusses any hit in broad biological terms, often with random disease links. The authors should mine their data more deeply and propose a model explaining the developmental logic of GC proteome progression.

- Data presentation needs improvement. The heatmaps are unreadable at the protein level. What are the top enriched proteins per timepoint? Have authors thought about volcano plots to indicate their most differentially expressed hits?

Minor edits:

Line 58 - "inappropriate connections" sounds like jargon. Also, did the authors consider synaptic pruning?

Line 91 - the sentence is unclear and needs revision.

© 2026. Published by The Company of Biologists under the terms of the Creative Commons Attribution License (https://creativecommons.org/licenses/by/4.0/).

Reviewer 2

This manuscript provides a valuable resource by profiling growth cone (GC) proteomes from the whole hippocampus (HP) and dentate gyrus (DG) across early postnatal development. The dataset covers a critical period for circuit refinement and will be useful for researchers studying axon development and hippocampal wiring. The study is well executed, and the classical GC fractionation method is applied consistently. The analysis and discussion of the data are comprehensive and highly scholarly. I support publication, provided the authors address the following methodological and interpretative concerns.

Major Comments:

1. Methodology While the Pfenninger-type fractionation is a classic method, it enriches for growth cone particles based on density but does not purify them. In the Western blot QC (Fig 2), the authors validated their fractionation protocol and wrote "we detect all GC associated proteins... in the F1 fraction and none of the other markers [PSD-95, Synaptophysin, MAP2]." However, the interpretation of "purity" is complicated by the choice of controls. The positive controls (TP and Syn) appear to be derived from adult tissue, whereas the fractionation (PHN, F1, F2) is performed on P4 tissue. The starting material for the gradient (PHN) shows barely detectable levels of PSD-95 or Synaptophysin, and therefore their absence in the F1 fraction does not confirm successful removal/purification. I think that the authors should address the identity of F1 fraction with more evidence or discussion. For example, how much proteins from this fraction are likely to originate from the growth cone? And will the difference in signal-to-background ratios (i.e. fewer growth cones in DG at P1 compared to P3 and P5) affect this proportion?

2. Origin and heterogeneity of GCs The GC fractions isolated from CA and DG inevitably contain multiple neuronal subtypes. And the relative abundance of these subtypes changes markedly between P1 and P5, with GCs of HP-resident neurons growing daily. This limitation should be stated clearly to guide appropriate interpretation. In this sense, the difference of F1 fractions of HP and DG may arise also from global DG maturation patterns and increased cellular heterogeneity, as well as from GC-intrinsic changes. This possibility should be addressed with more evidence or discussion.

3. Typographical issues
- "quadradic regression model" (Methods section), quadratic regression model?
- "conduced with STRING network analysis" (Cluster 4 description), conducted?
- "neuronal protection guidance" (Cluster 4 description), projection?
- "dataset identifier XXXX" (Data availability), XXXX?

First revision

Author response to reviewers' comments

Response to the reviewers Krause et al. 2026:

Thank you very much for the opportunity to revise our manuscript “Proteomic profile of hippocampal growth cones through early postnatal development” (MS ID#: dev.205342).

We thank the reviewers for their constructive feedback and suggestions, which helped us to improve the manuscript significantly. We believe that this revised version is strengthened and hope it is now suitable for publication in Development.

We addressed all comments and suggestions from the reviewers. Please find the comment-by-comment responses (in bold) below. Furthermore, we shortened the manuscript as much as possible without sacrificing clarity (highlighted in green). Changes based on reviewers’

© 2026. Published by The Company of Biologists under the terms of the Creative Commons Attribution License (https://creativecommons.org/licenses/by/4.0/).

comments are indicated in yellow.

Comments from the Reviewers:

Reviewer 1: SUMMARY OF THE ADVANCE MADE IN THIS PAPER AND ITS POTENTIAL SIGNIFICANCE TO THE FIELD

Krause et al. address an important question about local proteome dynamics in the growing axon during brain development. The authors isolate growth cones of entorhinal cortex neurons at three timepoints and analyze protein homogenates by mass spectrometry.

We thank Reviewer 1 for the detailed feedback and the valuable suggestions to improve our manuscript. We would like to clarify that mass spectrometry analysis was not performed specifically on growth cones of entorhinal cortex neurons, but rather on growth cones of the hippocampal and dentate gyrus subregions, which also include growth cones from entorhinal cortex fibers. Please find a detailed response to every comment below.

SUGGESTIONS TO AUTHORS

What is well done:

- I am convinced that proteomics is central for understanding neurodevelopment, especially given the dominance of transcriptomics. The authors should make a stronger point that their methodology stands out, and anchor it with seminal proteome work from the Macklis, Silver, Miller, Kraushar, Barna and Tarabykin labs.

As suggested, we strengthened the description of our methodological approach and added more of the suggested references (Lines 95-100). Unfortunately, we were not able include all suggestions due to the word limit of the article.

What needs revising:

- The abstract needs a full rewrite. It repeats results rather than offering a conceptual, thoughtful synthesis of the findings. Sentences such as "This earliest timepoint seems to be characterized by a high metabolic demand" should be removed, as they add no real information. A short explanation of EC layer 2 Reelin axons would also help readability.

We rewrote the abstract to lay a stronger focus on the conceptional aspects of our study and added a short explanation of the analysis performed on the EC Layer2 Reelin axons (Lines 12-28).

- Abbreviation use is inconsistent. Sometimes terms are defined and sometimes not. Please revise.

We revised all used abbreviations and introduced each abbreviation once in the main text (Lines 65, 68, 74-75, 82, 111, 161, 169, 176, 182, 603-605).

- The sentence "Notably, we identified multiple proteins containing nuclear localization sequences…" should be removed. It is speculative and unsupported. The presence of an NLS in a sequence does not establish function.

The sentence was removed during rewriting of the abstract.

- The references throughout need revision. For example, the mention of local protein expression (line 64-65) cites only a small fraction of relevant work. Please include newer evidence showing that local protein synthesis and degradation are key molecular drivers in development. The rationale of studying hippocampal growth cones is also not explained (line 68-69).

We expanded this section in the introduction and added more recent publications including Borisova et al., 2024; Harnett et al., 2022; and Veeraraghavan et al., 2026. (Lines 95-98). We are also clarifying why we study hippocampal growth cones in Line 90.

© 2026. Published by The Company of Biologists under the terms of the Creative Commons Attribution License (https://creativecommons.org/licenses/by/4.0/).

- Figure 1: zoom in on the quantified structures shown in the bar graphs in panel B; include these as insets in panel A. Panel C needs arrowheads. The legend must specify what is plotted, what the bars represent (mean?), and what the error bars denote. Report the statistical tests used.

We clarified the analysed area for panel B in Figure 2 by adding a schematic overview of the subregions of interest in the same panel. Supplement Fig. S1 also contains a representative image with the indication of pixels above the threshold used to detect the fluorescence signal in the subregions of interest. We added the arrow heads to panel C and improved the figure caption to specify better the depicted results and included a cross- reference to Fig. S1 (Lines 1142-1156). In addition, we changed the labelling of the fluorescent signal from the entorhinal fibers from GCamP6 to GFP to avoid any confusion between the detection of the GFP part of GCamP6 with the help of immunohistochemistry and active calcium signalling events (Lines 129-130, 136, 584-584, 605, 1144). More detailed explanation see below.

- The mouse model is not described. Please explain the rationale for the transgenic line and what this line is. Other than visualizing GCs, it does not really serve the purpose - the reader is inclined to think some sort of fluorescence-based isolation would follow.

We added a more precise description of the mouse lines used for the Characterization of the critical time window of the invasion of the Hippocampus (Lines 125-127). In addition, we changed the title of the first section of the results to clarify the purpose of the immunohistochemical analysis of the early postnatal days (Lines 117-118). Finally, we adjusted the transition to the following results to avoid the misconception that we are going to use a fluorescent based isolation in the following experiments (Line 148).

- Line 114 is unclear. The chosen timepoints appear arbitrary. Please explain the rationale, including the "critical window" referenced.

We rewrote this section to clarify why this critical time window is important for the development of the hippocampus (Lines 120-123).

- Line 115 - remove "elegant."

We removed “elegant” (Line 122).

- What is the contribution of Odz3-expressing Reelin cells to EC neuronal cell types, and why are these cells important?

Odz3 enhancer expressing Reelin cells are the Layer 2 cells in the entorhinal cortex. Those cells form the major input of the entorhinal cortex to the hippocampus and connect to the subregions CA3 and Dentate Gyrus. To clarify this connection, we improved the description of the mouse line (Lines 125-127) and rewrote the corresponding part in the introduction (Lines 78-79).

- Line 121 - why is the GCaMP6 line used to label axons? Could lower fluorescence at P1 reflect reduced Ca2+ waves rather than lack of invasion?

For the characterization of the invasion of the hippocampus by Reelin-positive Layer 2 entorhinal fibers we tested available mouse lines at our institute. Available reporter mouse lines were a TetO-GFP mouse line and the TetO-GCamP6 mouse line used in the study. The TetO-GFP line unfortunately did not express the GFP reporter in early postnatal days, while the TetO-GCamP6 line showed a clear expression of GCamP6 in the same time window.

It is correct that GCamP6 is a calcium sensor and normally shows increase in fluorescence after calcium binding. However, in the described experiments we are using fixed tissue and detect the presence of the GCamP6 protein by using immunohistochemistry against the GFP part of GCamP6. Thus, the detected fluorescent signal for the labelled axons reflects the presence of the protein itself, rather an active calcium signal. We changed the labelling of our immunohistochemical staining and analysis from GCamP6 to GFP to clarify what we are truly detecting in the experiment. (Lines 129-130, 136, 584-584, 605, 1144).

© 2026. Published by The Company of Biologists under the terms of the Creative Commons Attribution License (https://creativecommons.org/licenses/by/4.0/).

- Line 129 - avoid wording like "well-known."

Removed (Line 135).

- Line 131 - the listed numbers of GCs counted are confusing. Is this per animal, per hippocampus? What does this number represent?

We rewrote this part to make it more comprehensible. The total number of GCs counted (Fig. S1C) represents the number of GCs counted over all animals and sections analysed (Lines 137-139).

- Given that EC laminae project to different hippocampal regions (ML, SML), could the target region itself explain local proteome differences? If synaptic targets differ, presynaptic composition would be expected to differ as well. What is the influence of neuronal fate on growth cone composition?

These questions are one of the major driving factors that led to this study. We agree that the target region can strongly influence the local proteomic differences, but at the same time the influence of the neuronal fate cannot be ignored for the proteomic composition of GCs. Our current study shows that the proteomic composition is clearly changing during the development and when GCs are reaching their final target. However, at the moment and on basis of the presented dataset we cannot disentangle what the effects of the target region on the GCs are and what the effects of the development itself are. This is the major limitation of this study, and we have better clarified this in the text (Lines 436-438, 531- 537). In future studies, we would like to address this question in more detail, by focussing only on GCs coming from one specific cell type and studying the proteomic changes over time and specific for a target region.

- Figure 2: the microdissection validation is unclear. This step is critical to confirm that the proteomics reflect region-specific material. qRT-PCR alone does not validate this (especially with the observed data spread). Why not perform histology or ISH on dissected regions? In panel C, why is no presynaptic marker included, given that it should be enriched in GC fractions? Also, F1 is used for proteomics, yet the WB shows enrichment of GAP-43 in F2. Please clarify.

We agree that the validation of the dissection is critical to ensure that the proteomic data reflects region-specific material. Before working with brain tissue of young animals, we implemented the dissection technique on adult tissue. To confirm the quality of our dissection we used RNA and protein extracts from the dissected tissue and tried to detect the CA and DG markers published by Hagihara et al. 2009. The marker tested here in qRT- PCR and DNA microarray were tryptophan 2,3-dioxygenase (Tdo2) and desmoplakin (Dsp), and Ammon's horn enriched genes, Meis-related gene 1b (Meis 2) and TYRO3 protein tyrosine kinase 3 (Tyro3). We could not see any differences between the CA and DG protein extracts when running western blots on the dissected sample (s. below), while the difference between the CA and DG tissue was clearly detectable on the RNA level. This is likely a reflection of the proteins been present also in the neuropil while the RNA molecules for these markers are mainly restricted/enriched in the soma. Note that based on the GAPDH loading control show that the 67075 DG sample has a higher protein load than the other lane. Thus, all larger signals for the marker proteins have to be associated with the higher protein load and are not due to a higher expression.

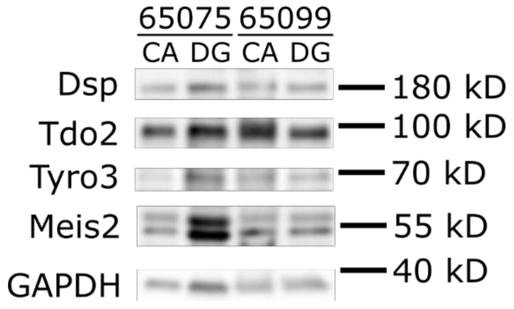

© 2026. Published by The Company of Biologists under the terms of the Creative Commons Attribution License (https://creativecommons.org/licenses/by/4.0/).

Figure 1: Western blot for dissection control on adult tissue of two animals (65075 and 65099). Loaded are 5 µg of total protein extracts generated with Machery-Nagel RNA/Protein extraction kit. Dsp, Tdo2, Tyro3, and Meis2 are published markers to confirm the dissection of DG and CA. Dsp and Tdo2 are DG-associated and Tyro3 and Meis2 are CA associated.

The Western blot could suggest that either the dissection was not specific enough or that the proteins used as marker were non-specifically restricted to the subregions. We therefore decided to perform qRT-PCR and our results recapitulated those of Hagihara et al. 2009, confirming the validity of our dissecting method. Another advantage to qRT-PCR is that we can use the ISH data of the Allen brain atlas (atlas.brain-map.org) to find suitable genes to control our dissection, as not all the markers used for adults' tissue were working for pup tissue. Please find the respective Allen data for the used markers below. We respectfully disagree that the qRT-PCR results with the observed data spread is not confirming the quality of our dissection, because 5 out of 6 tested markers show highly significant differences between the CA and DG tissue after dissection (Manuscript Fig. 2 B).

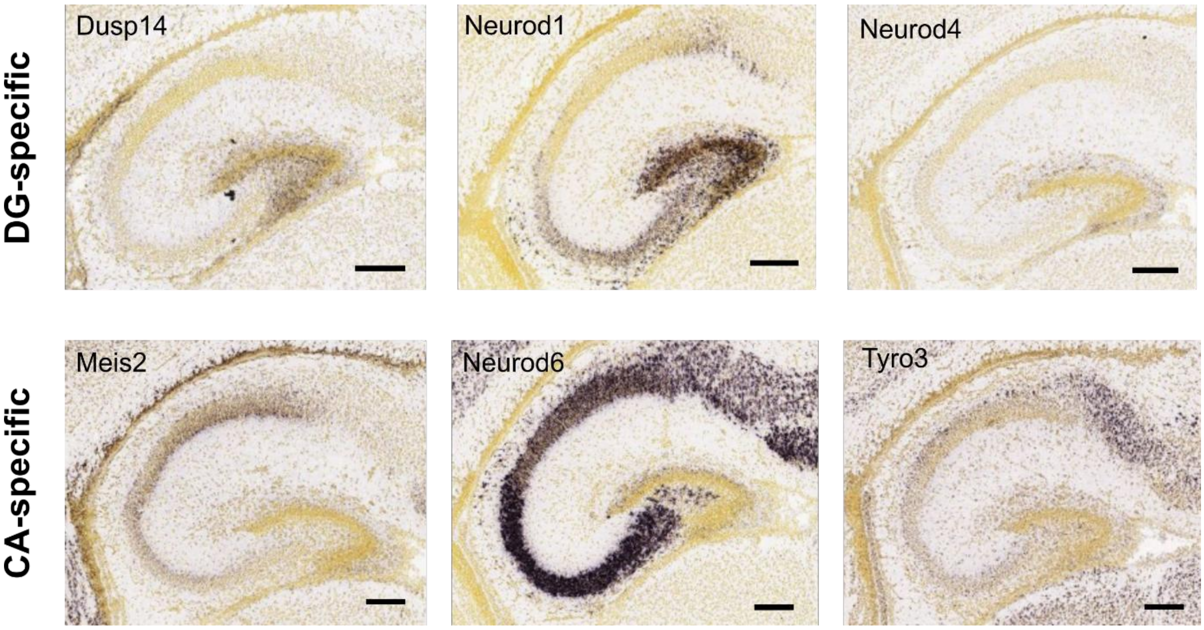

Figure 2: Allen IHC data at P4 for genes used in qRT-PCR. The top row shows the DG-specific genes and the lower row the CA-specific ones.
Dusp14 https://developingmouse.brain-map.org/experiment/show/100084360
Neurod1 https://developingmouse.brain-map.org/experiment/show/100054896
Neurod4 https://developingmouse.brain-map.org/experiment/show/100056053
Meis2 https://developingmouse.brain-map.org/experiment/show/100072268
Neurod6 https://developingmouse.brain-map.org/experiment/show/100081462
Tyro3 https://developingmouse.brain-map.org/experiment/show/100056093

Our dissection approach is performed on fresh tissue and the whole hippocampus as one. Please see the images of a dissected hippocampus and the subsequent dissection of CA and DG in the image below. For the GC extraction we are either using the hippocampus as depicted in Figure 3B or the DG as shown in Figure 3D.

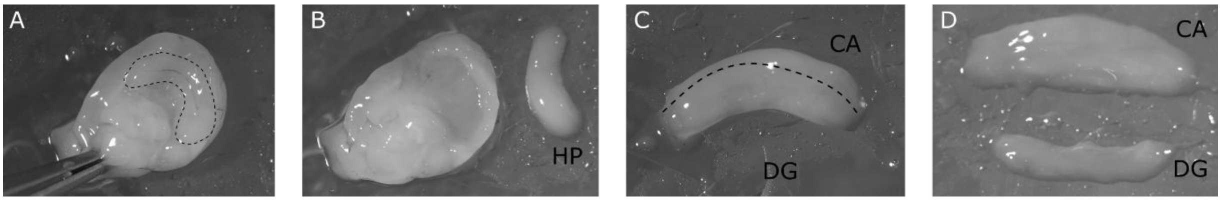

© 2026. Published by The Company of Biologists under the terms of the Creative Commons Attribution License (https://creativecommons.org/licenses/by/4.0/).

Figure 3: Example images for the dissection of the DG from the hippocampus of a P12 animal. ***A*** *shows the one hemisphere of a mouse brain after removing the white matter. The exposed hippocampus is indicated with the dashed area. The dissected hippocampus from the cortex is shown in* ***B****.* ***C*** *shows the isolated hippocampus from the cortex with its medial side up. The dashed line indicated the visible line between CA and DG. Note the DG only forms approximately 1/4th of the total hippocampal tissue. Not visible from this view is, that the CA regions are also laying below the DG.* ***D*** *shows the separated CA regions from the DG.*

Performing histology or ISH would require sections of fixed tissue that we are not generating with our approach and that would not, in our opinion, validate the dissection, but rather the specificity of the markers used. We therefore decided to work with the fresh tissue and a method that allows us to verify the dissection reliably and with as little extra processing steps as possible.

Fig.2C includes the presynaptic marker Synaptophysin, which is highly abundant in adult tissue. This marker is used to exclude that we have mature structures such as presynaptic tissue in our preparation. GCs are the tip of a growing axon and are considered to be different from presynapses. Thus, Synaptophysin is not expected be enriched in our GC preparation.

As correctly noted, the F1 fraction is used for proteomic data. Pfenninger et al., 1983 and Lohse et al., 1996 showed in their studies that GCs particles are enriched on the border between 0.32 M sucrose and 0.75 M sucrose or 0.32 M sucrose and 0.83 M sucrose on a sucrose density gradient, respectively. Both papers call this fraction F1. Gap-43, though considered to be a GC marker, is also a cytoplasmic protein present in other parts of the neuron and not only located in GCs. As such, it is expected to be detectable also in the F2 fraction (Lines 169-171).

- The biochemical preparation of growth cones is confusing. Can the authors visualize GCs by microscopy and exclude other subcellular contaminants? If not, the fraction should be named differently.

For the preparation of GCs we followed the well-established protocol by Lohse et al., 1996. This approach is the gold standard for GC preparation. We used the common nomenclature of the fractions based on this method. The GC particles generated with this method have an average size between 0.3-0.9 µm (Poulopoulos et al. 2014) and were well characterized by electron microscopy in Pfenninger et al. 1983 and Lohse et al., 1996. Here they show that the majority of particles in the F1 fraction originated from GCs. Other subcellular structures are separated based on their density on the sucrose gradient and are collected in the F2 ("trash") fraction. Furthermore, based on these two studies the preparation of GCs can contain free cytosolic proteins that might interfere with any kind of analysis. Therefore, we introduced a washing step of our preparation to ensure that we enrich the GCs in our samples. Please see Fig. S2 that confirms that the washing step we are using at the end of our GC preparation increases the amount of GCs per 1 µg of total protein compared to an unwashed preparation (see below), as proteins associated with GCs are more easily detected in the washed compared to the unwashed GC preparation. However, we cannot completely exclude that our preparation contains small amounts of random contaminants. We revised our description of the preparation to ensure clarity on this (Line 159-164).

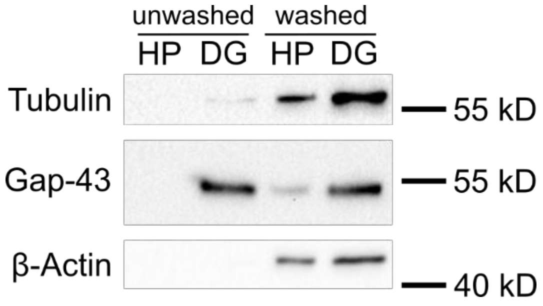

Fig. S1: Western blot control of unwashed and washed GC F1 fraction from HP and DG at P5. Loaded were 1 µg of protein per lane. GC associated proteins Tubulin, Gap-43, and ß-Actin can be clearly detected

© 2026. Published by The Company of Biologists under the terms of the Creative Commons Attribution License (https://creativecommons.org/licenses/by/4.0/).

in the washed preparations and only to some extent or not at all in the unwashed preparations confirming that the washed F1 fractions used in our study have an increased amount of GCs compared to unwashed preparation.

- Line 172 - provide the exact ages of animals used for each fraction. Are all fractions in Fig. 2C age-matched? If not, the comparison is not meaningful.

We added the age of all animals to the figure caption (Lines 1158, 1162, 1165).

- Line 184 - the sample description reads as if one animal generated five technical replicates. This design is not acceptable; replicates should be biological, from age-matched animals. How does sex contribute to sample preparation? How many male and female animals were used?

We rewrote this part of the text to clarify that we sampled five independent animals for each time point (Lines 176-178). For one sample both hippocampi of one animal were pooled. P1: 5 animals for HP-GCs, P3: 5 animals for HP-GCs, and P5: 5 animals for HP-GCs. In total 15 animals used to generate all samples for HP-GCs.

For DG-GCs we sampled accordingly but only used the DG for the GC preparation. This were another 15 animals, distributed as for the HP-GCs, 5 for each time point (Lines 250- 253).

Animals of either sex were used, but it was not checked during sample preparation. We clarified this in the manuscript in Line 539 and 563.

- Figure 3: did the authors validate the proteomic findings? For example, if P1 GCs are enriched for mitochondria (line 208) as compared to other stages, you could image mitochondria at P1, P3 and P5, similar as done on Fig. 1.

Based on this suggestion we performed western blot analysis to validate our proteomic findings on SDHA a mitochondrial marker protein and HNRPH2, a RNA metabolic protein. Both proteins are highly abundant at P1 and are less abundant at P5. We chose western blots instead of IHC as mitochondria are present in all parts of the cell and it might bedifficult to disentangle the signal from other cellular structures that are around the GCs. SDHA is a widely used mitochondria marker and provides a direct readout on the amount of mitochondria in a sample.

For the western blot we prepared total protein extract and GCs from the hippocampus at P1 and P5 and loaded 1 µg of total protein per lane (see below).

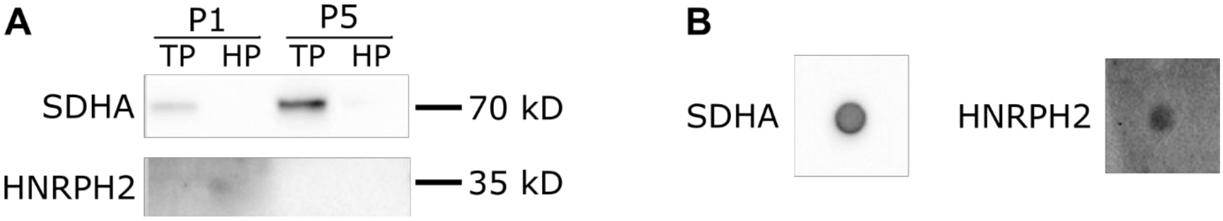

*Figure 4:**A** Western blot analysis of SDHA and HNRPH2 on total protein extract (TP) and GC from the hippocampus at P1 and P5. **B** shows dotplots to control for primary antibody functionality on adult hippocampal total protein extract.*

For HNRPH2 a band around 35 kD was expected, but we were not able to detect any signal for this protein. The dot blot control, performed on adult hippocampal tissue, shows that the primary antibody is working, thus, we assume that the HNRPH2 abundance is below the detection limit at young age. For SDHA we see bands with increasing intensity in the total protein extract. This indicates that more mitochondria are present at P5 compared to P1. This result is expected for maturing tissue. For the GC samples no signal for SHDA was detectable, which we again attributed to abundance below the detection limit of the western blot approach. Between the tissue extract and GCs, we observe two opposing trends for SDHA. With increasing maturation, mitochondria are decreasing in the GCs (based on proteomic data) while at the same time mitochondria increase in the total tissue. At later developmental stage, we

© 2026. Published by The Company of Biologists under the terms of the Creative Commons Attribution License (https://creativecommons.org/licenses/by/4.0/).

can expect an increase in the number of mitochondria to keep up with higher energy demand of mature neurons. In contrast, as suggested in the text, maturing GCs have a decreasing energy demand, as they reach their target region and start to transition into forming a synapse.

Furthermore, we wanted to validate some of the differences we observed in our proteomic data at P5 between the hippocampus and DG. Thus, we chose candidates to be tested based on either of the following criteria: upregulated in any of the time-series analysis or only identified at P5 in HP-GCs or DG-GCs. For this validation we used a total protein extract of the hippocampus at P5, HP-GCs, and DG-GCs (both generated from P5 animals) and loaded 1 µg per lane. We tested the proteins WASF2 (only identified for HP P5), Nova2 (DG upregulated, single time series), and C1qA (only identified for DG P5). The following figure shows the results of the western blots.

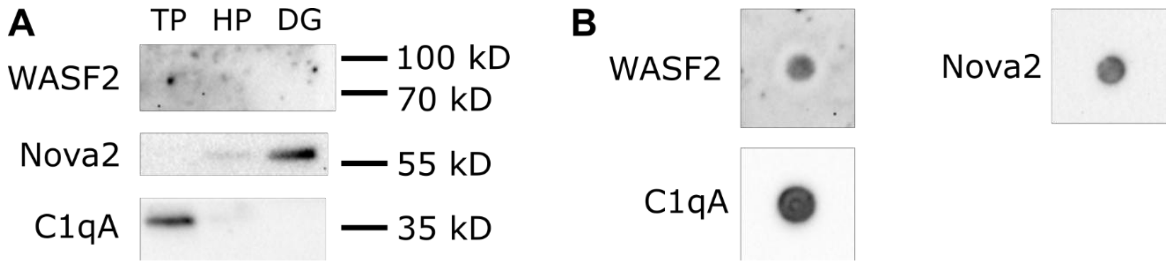

*Figure 5: Western blot analysis for selected proteins at P5. **A** Loaded are hippocampal total protein extract (TP) from P5 animas, HP-GCs (HP), and DG-GCs (DG). **B** Dot blots for primary antibody control on adult total protein extract.*

For WASF2 (ca.90 kD) no clear signal could be detected on the western blots potentially indicating that the abundance of WASF2 is too low at P5 to be detectable on a Western blot. For C1qA, only a signal in the total protein extract was visible but not in the GC preparations. We attribute this to low abundance of the protein in the GC sample. Nova2 shows a strong signal in DG-GCs, reflecting the results observed in our proteomic data analysis.

Summarizing, we can conclude that validating GCs proteomic data by western blots is limiting, as the amount of protein in GCs is often below detection level. This is the case at least for the majority of the chosen proteins we tested, as no signal could be detected in the GC preparations. Only Nova2 showed the expected results and confirms a strong signal for P5 specifically DG-GCs that corresponds to our observations in the proteomic data. Interesting, the absence of Nova2 signal in the TP could suggest that some of the proteins detected in the GC are not widely expressed in the cell, but rather specifically enriched in the GC.

- Line 215 - references again need revision. For instance, Harnett et al., 2022 should be cited for tRNA in development.

We added the suggested citation (Line 205).

- Figure 3E: why are only some proteins shown on the heatmap instead of full cluster contents? GO terms should be run for each cluster.

The proteins shown in the heatmap in Fig.3 are the ones that were identified as significant in the single time series analysis for HP-GCs. We added this information in the figure caption (Lines 1177-1178) to clarify why those proteins were plotted in the heatmap in Fig. 3E.

The GO term analysis is not run for the significant proteins of the time series analysis but for all proteins that were identified as exclusive of one time point indicated in the Venn diagram in Fig3. D.

- The Fig. 3 data are problematic. The GC proteomes are not normalized to global protein levels in the region or to nuclear fractions. This likely explains the apparent spike in protein abundance at P3

© 2026. Published by The Company of Biologists under the terms of the Creative Commons Attribution License (https://creativecommons.org/licenses/by/4.0/).

across clusters. This should be considered in the interpretation. Including EC proteomes at P1, P3 and P5 might help.

The proteomic data presented in this study is normalized to the amount of input tissue. For each sample both hippocampi or DGs were used to generate GCs and the whole GC preparation was subjected to proteomic analysis. To ensure comparability between the samples the raw data was interpreted using the label free quantification (LFQ) approach provided by MaxQuant (MaxLFQ). We are not sure about what the reviewer means when mentioning the "apparent spike in protein abundance". One possible interpretation of the comment is that the reviewer is suggesting that the increase of GC numbers between P1 and P3 introduced the spike in the averaged abundance plots. However, we would like to point out that the averaged abundance plots only show the data of the 15 proteins that were identified as significantly different in their expression profile in the single time series analysis. One of the basic assumptions for LFQ normalization is that most of the proteins in the dataset remain roughly constant across samples, we are convinced that this is met in our dataset, as the majority of proteins are not showing the described peak at P3.

In addition, we changed the term abundance to log LFQ intensities throughout the manuscript to generate more clarity on the presented data.

- The results section needs reconceptualization. Pages 9-13 read as a loose walk-through of GO terms rather than a coherent narrative. What was the aim of the analysis? What was the original aim of the authors? Some experimental validation of highlighted processes (e.g., line 239-240) would help and I strongly encourage authors to follow-up.

Our lab is interested in understanding how different cell types contribute to the development of the hippocampal circuit. With the generated proteomic dataset, we want to cover the early postnatal days and understand what biological processes are the most critical ones. Since we are not disentangling the GCs on a cellular level but rather study GCs extracted from the whole hippocampus or DG as one population, we are limited by this rather crude scale. However, this still allows us to identify the most relevant biological processes that are crucial for the early postnatal days independent of the cell type and highlight that the proteins present in GCs change very dynamically in early development in a sub-region specific way. We improved the result section by heavily rewriting it, especially by focusing on shortening the sections to give a more coherent reading experience and improving transitions between the different subsections (all highlighted in green and Lines 176, 249-250, 347-355, 357-361, 436-638). Future work will focus on the most interesting findings of this paper and will allow to specifically validate the highlighted processes in the context of a single cell type, but this would require extensive work which we feel as beyond the scope of this study.

- Part 6 lacks focus and feels out of place. It discusses any hit in broad biological terms, often with random disease links. The authors should mine their data more deeply and propose a model explaining the developmental logic of GC proteome progression.

Our intention was to give a broad overview on all proteins in our dataset that are associated with neurological disorders and neurodegenerative diseases; however, we understand that this might appear too unfocused. Therefore, we changed the section and focus now on diseases that are associated with the time regulated proteins to in the HP- GCs and DG-GCs dataset (new Table 1, Lines 459-462). We included the extended list of disease associated proteins in the supplement as Table S2 and included a section on relevance of development for neurological disorders and neurodegenerative diseases in the introduction (Lines 101-107).

- Data presentation needs improvement. The heatmaps are unreadable at the protein level. What are the top enriched proteins per timepoint? Have authors thought about volcano plots to indicate their most differentially expressed hits?

We agree that the proteins indicated in the heatmaps are difficult to read. To improve the data presentation, we increase the heatmap in Fig.4 to the maximal height possible and moved the depiction of the average intensities of the detected clusters to the supplement Fig. S4.

© 2026. Published by The Company of Biologists under the terms of the Creative Commons Attribution License (https://creativecommons.org/licenses/by/4.0/).

Furthermore, we moved the heatmap originally presented in Fig. 6 to the supplement as new Fig. S5. Again, we increased the height of the heatmap to the possible maximum to improve readability.

Depicting the results of our time series analyses in volcano plots will not increase the comprehensibility of the data. Volcano plots will only allow us to represent two of the three time points we are analysing. This depiction might lead to misinterpretations of the data, as some proteins might appear significantly different between two timepoints, but are not changed between the other two time points. Volcano plot representation of the time series analyses will include at least three separate plots that needs to be interpreted together to convey the same information as the heatmap provides. The maSigPro time series analysis is specifically design for the parallel evaluation of multiple time points and provides the advantage of a clear presentation of the results as heatmap and average expression profiles (please refer to Dumrongprechachan et al. 2022, eLife).

Minor edits:

Line 58 - "inappropriate connections" sounds like jargon. Also, did the authors consider synaptic pruning?

We revised the whole paragraph and shortened it. In addition, we removed the word inappropriate or replaced it with “unspecific” (Lines 54, 58). In this context, we wanted to emphasise that GCs are leading the way to the target region and are not randomly connecting to every possible target on their way. We agree that synaptic pruning is one of the critical steps in circuit development, however most synaptic pruning occurs in postnatal week two and three in the hippocampus and is less critical for the pathfinding process of the GCs themselves (Paolicelli et al., 2011, Science).

Line 91 - the sentence is unclear and needs revision.

We changed the sentence to clarify our experimental procedure (Lines 108-109).

Reviewer 2: This manuscript provides a valuable resource by profiling growth cone (GC) proteomes from the whole hippocampus (HP) and dentate gyrus (DG) across early postnatal development. The dataset covers a critical period for circuit refinement and will be useful for researchers studying axon development and hippocampal wiring. The study is well executed, and the classical GC fractionation method is applied consistently. The analysis and discussion of the data are comprehensive and highly scholarly. I support publication, provided the authors address the following methodological and interpretative concerns.

We thank Reviewer 2 for the positive feedback on our manuscript. We addressed all comments in the revised manuscript. Please find detailed answers to each comment below.

Major Comments:

1. Methodology

While the Pfenninger-type fractionation is a classic method, it enriches for growth cone particles based on density but does not purify them. In the Western blot QC (Fig 2), the authors validated their fractionation protocol and wrote "we detect all GC associated proteins... in the F1 fraction and none of the other markers [PSD-95, Synaptophysin, MAP2]." However, the interpretation of "purity" is complicated by the choice of controls. The positive controls (TP and Syn) appear to be derived from adult tissue, whereas the fractionation (PHN, F1, F2) is performed on P4 tissue. The starting material for the gradient (PHN) shows barely detectable levels of PSD-95 or Synaptophysin, and therefore their absence in the F1 fraction does not confirm successful removal/purification. I think that the authors should address the identity of F1 fraction with more evidence or discussion. For example, how much proteins from this fraction are likely to originate from the growth cone? And will the difference in signal-to- background ratios (i.e. fewer growth cones in DG at P1 compared to P3 and P5) affect this proportion?

© 2026. Published by The Company of Biologists under the terms of the Creative Commons Attribution License (https://creativecommons.org/licenses/by/4.0/).

We agree that the original method implemented by Pfenninger at al., 1983 for GC preparation/enrichment also contains a certain amount of soluble protein ("protein contaminants"). To reduce the amount of these contaminants we introduced a washing step before western blot analysis. To show that this washing step increases the quality of our GC preparation we performed a Western blot on unwashed and washed GC preparations of hippocampus and DG at P5 and included this analysis in the new supplement figure Fig.S2. We detected tubulin, Gap-43 and ß-Actin for those samples and could show that the washing increases the signal for those proteins in the washed preparations. Based on this we conclude that in the fraction of GC in the washed samples per 1 µg protein is significantly higher than in the unwashed fractions (see below).

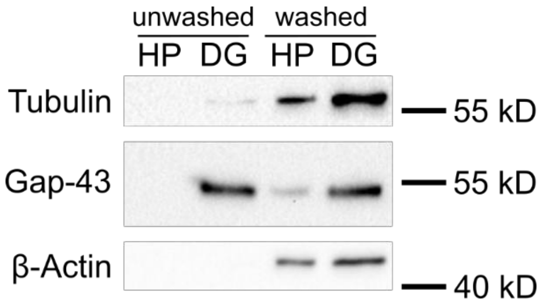

Fig. S2: Western blot control of unwashed and washed GC F1 fraction from HP and DG at P5. Loaded were 1 µg of protein per lane. GC associated proteins Tubulin, Gap-43, and ß-Actin can be clearly detected in the washed preparations and only to some extent or not at all in the unwashed preparations confirming that the washed F1 fractions used in our study have an increased amount of GCs compared to unwashed preparation.

Pfenninger et al. states that their preparation contains around 70% of protein contamination from the homogenate. By applying the washing step as indicated in the revised manuscript, we estimate that this contamination is reduced to less than 5 % of the sample. We included the contamination aspect more clearly in the manuscript and rewrote our interpretation of the original western blot (Lines 162-164). In addition, we adjusted the used reference for the GC preparation because we are using sucrose concentrations that were published by Lohse et al., 1996 (Line 159). In this paper the authors discuss that the sucrose concentration used in their study improves the quality of the GC preparation and reduces the amount of contaminants. Taking this and our washing step together into account, we are convinced that we remove the majority of protein contaminants for the western blot analysis.

In addition, when preparing the GC samples for proteomic analysis, we are filtering each of our preparation on a filter with the pore size of 0.1 µm and wash each sample with 4 mL of PBS after it was filtered. This is an even more rigorous washing than applied for the western blot analysis and should reduce the protein contamination to a minimal level in the proteomic data analysis. We clarified this procedure in the main text (Lines 162-164, 179). and the method part (Lines 678-682, 684-685).

2. Origin and heterogeneity of GCs
The GC fractions isolated from CA and DG inevitably contain multiple neuronal subtypes. And the relative abundance of these subtypes changes markedly between P1 and P5, with GCs of HP-resident neurons growing daily. This limitation should be stated clearly to guide appropriate interpretation. In this sense, the difference of F1 fractions of HP and DG may arise also from global DG maturation patterns and increased cellular heterogeneity, as well as from GC- intrinsic changes. This possibility should be addressed with more evidence or discussion.

To ensure that the reader is aware of this limitation we revised the first paragraph of the discussion and provided an overview of ongoing developmental processes in our studied time window (Lines 465-472). Furthermore, we addressed the heterogeneity of the sample in Line 148 and Lines 501-502. We include the aspect of DG granular cell neurogenesis in Lines 468-470 but were unfortunately not able to include an in-depth discussion of this process and

© 2026. Published by The Company of Biologists under the terms of the Creative Commons Attribution License (https://creativecommons.org/licenses/by/4.0/).

potential effects on the DG-GC dataset because of the word limitation of the manuscript.

3. Typographical issues
- "quadradic regression model" (Methods section), quadratic regression model?
- "conduced with STRING network analysis" (Cluster 4 description), conducted?
- "neuronal protection guidance" (Cluster 4 description), projection?
- "dataset identifier XXXX" (Data availability), XXXX?

We corrected the typos (Lines 323, 739, 1189) and added the missing information links to Github and ProteomeXchange (Lines 731, 750).

Second decision letter

MS ID#: dev.205342R1

MS TITLE: Proteomic profile of hippocampal growth cones through early postnatal development

AUTHORS: Maike Krause; Kamilla Aase Kronberg; Paulo J. B. Girão; Lara Sophie Strohmeier; Giulia Quattrocolo

Dear Dr Quattrocolo,

I have now received all the referees reports on the above manuscript, and have reached a decision. The referees' comments are appended below.

The overall evaluation is positive and we would like to publish a revised manuscript in Development, provided that the referees' comments can be satisfactorily addressed. Please attend to all of the reviewers' comments in your revised manuscript and detail them in your point-by-point response. Specifically please address concerns about the abstract and need for supplemental material for Fig 2. Please send us a point-by-point response indicating your plans for addressing the referees' comments, and we will look over this and provide further guidance.

Reviewer 1

The authors have substantively addressed most of my previous comments and the manuscript has improved considerably. I appreciate the effort that went into revising the conceptual framing, clarifying the rationale for the developmental timepoints, improving the description of the mouse line and growth cone preparation, and more explicitly discussing the limitations of the approach. Overall, the revised manuscript is much clearer and stronger.

My main remaining concern is the abstract. I still feel that it is poorly written and does not yet reflect the improved quality of the manuscript. For example, the sentence "While Hippocampal growth cones undergo a rapid transition, from RNA-related processes to synapse formation, Dentate Gyrus growth cones maintain an exploratory proteomic profile for longer, suggesting delayed maturation, consistent with later ingrowth of entorhinal cortex fibers" is difficult to follow. The comma placement interrupts the logic of the sentence, the capitalization of anatomical terms is inconsistent, and it is unclear what is meant by an "exploratory proteomic profile." It is also not obvious why maintaining such a profile would necessarily suggest delayed maturation. This is just one of the many sentences in the abstract which are problematic and personally would discourage me from engaging with this paper in depth.

I strongly encourage the authors to revise the abstract once more. The manuscript itself has become much more coherent, but the abstract still reads as a list of loosely connected result statements rather than a clear conceptual synthesis of the study. A more polished abstract would

© 2026. Published by The Company of Biologists under the terms of the Creative Commons Attribution License (https://creativecommons.org/licenses/by/4.0/).

help the paper make a stronger first impression and better reflect the improvements made during revision.

Also, please provide the "uncropped" original Western blotting results used for Fig. 2 in the supplementary material.

Reviewer 2

The authors have addressed the major methodological and interpretative concerns raised in the initial review. In particular, the limitations related to sample heterogeneity and growth cone enrichment are now more clearly acknowledged. While some aspects of the fraction purity remain difficult to quantify, this does not detract from the overall value of the dataset. I support publication of the revised manuscript.

Second revision

Author response to reviewers' comments

Response to the reviewers Krause et al. 2026:

We thank you and the reviewers for the time invested in providing valuable feedback on our manuscript “Proteomic profile of hippocampal growth cones through early postnatal development” (MS ID#: dev.205342).

The comment-by-comment responses can be found in bold below. We have addressed the comments from Reviewer #1 and highlighted the changes in yellow.

Comments from the Reviewers:

Reviewer 1: The authors have substantively addressed most of my previous comments and the manuscript has improved considerably. I appreciate the effort that went into revising the conceptual framing, clarifying the rationale for the developmental timepoints, improving the description of the mouse line and growth cone preparation, and more explicitly discussing the limitations of the approach. Overall, the revised manuscript is much clearer and stronger.

We thank Reviewer 1 for their feedback, and we are glad they find the manuscript stronger and considerably improved.

My main remaining concern is the abstract. I still feel that it is poorly written and does not yet reflect the improved quality of the manuscript. For example, the sentence "While Hippocampal growth cones undergo a rapid transition, from RNA-related processes to synapse formation, Dentate Gyrus growth cones maintain an exploratory proteomic profile for longer, suggesting delayed maturation, consistent with later ingrowth of entorhinal cortex fibers" is difficult to follow. The comma placement interrupts the logic of the sentence, the capitalization of anatomical terms is inconsistent, and it is unclear what is meant by an "exploratory proteomic profile." It is also not obvious why maintaining such a profile would necessarily suggest delayed maturation. This is just one of the many sentences in the abstract which are problematic and personally would discourage me from engaging with this paper in depth.
I strongly encourage the authors to revise the abstract once more. The manuscript itself has become much more coherent, but the abstract still reads as a list of loosely connected result statements rather than a clear conceptual synthesis of the study. A more polished abstract would help the paper make a stronger first impression and better reflect the improvements made during revision.

We have now rewritten the abstract and hope the reviewer find it more engaging and coherent (Lines 12-25).

© 2026. Published by The Company of Biologists under the terms of the Creative Commons Attribution License (https://creativecommons.org/licenses/by/4.0/).

Also, please provide the "uncropped" original Western blotting results used for Fig. 2 in the supplementary material.

Uncropped Western blotting results are added to Fig. S2 (panel B), and we included the reference in the manuscript (Lines 162-163). Please be aware that we are cutting the Western blot membrane horizontally to maximize the number of proteins detectable per blot while at the same time using as little sample as possible.

Figure 1 below shows the schemes we used for cutting the membranes of the Western blot shown in Fig. 2C and Fig. S2B. Please note that Gap-43 and b-Actin are detected in duplex, because Gap-43 is a rabbit antibody and b-Actin is a mouse antibody and both proteins are very close to each other on the Western blot membrane. For the duplex detection we incubated with both primary antibodies at once and subsequently incubate with the secondary antibodies separately including a detection step after each secondary antibody incubation. During each detection, we collect the bioluminescence signal until the first band reaches saturation. We detect b-Actin first, because the overall signal for b-actin is much lower than the Gap-43 signal, and then the Gap-43 signal. The lower signal for b- actin is also the reason why no b-actin bands are shown in the Gap-43 detection. The signal for Gap-43 reaches saturation before any b-actin signal is visible at all.

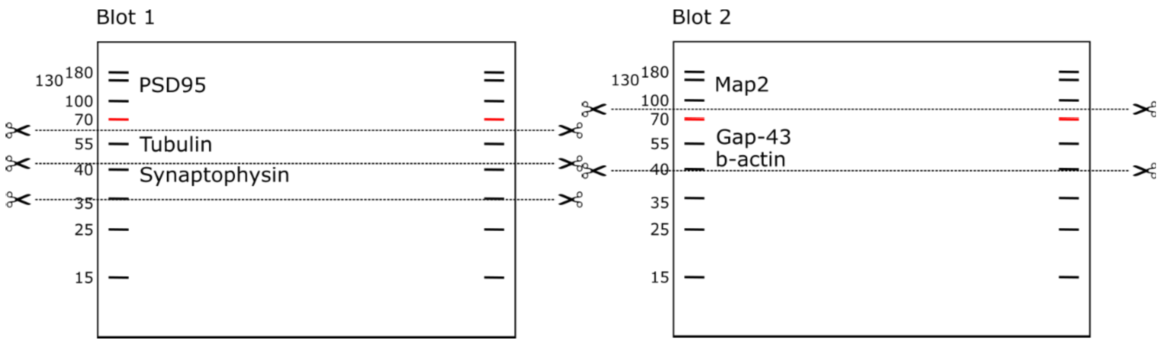

Figure 1: Cutting schema for the western blot membranes shown in Fig. 2C.

Reviewer 2: The authors have addressed the major methodological and interpretative concerns raised in the initial review. In particular, the limitations related to sample heterogeneity and growth cone enrichment are now more clearly acknowledged. While some aspects of the fraction purity remain difficult to quantify, this does not detract from the overall value of the dataset. I support publication of the revised manuscript.

We thank Reviewer 2 for the positive feedback on and the support for publication of the revised manuscript.

Third decision letter

MS ID#: dev.205342R2

MS TITLE: Proteomic profile of hippocampal growth cones through early postnatal development

AUTHORS: Maike Krause; Kamilla Aase Kronberg; Paulo J. B. Girão; Lara Sophie Strohmeier; Giulia Quattrocolo

Dear Dr Quattrocolo,

I am happy to tell you that your manuscript has been accepted for publication in Development, pending our standard publication integrity checks.

© 2026. Published by The Company of Biologists under the terms of the Creative Commons Attribution License (https://creativecommons.org/licenses/by/4.0/).
